# Power Conversion and Its Efficiency in Thermoelectric Materials

**DOI:** 10.3390/e22080803

**Published:** 2020-07-22

**Authors:** Armin Feldhoff

**Affiliations:** Institute of Physical Chemistry and Electrochemistry, Leibniz University Hannover, Callinstraße 3A, D-30167 Hannover, Germany; armin.feldhoff@pci.uni-hannover.de; Tel.: +49-511-762-2940

**Keywords:** thermoelectrics, power conversion, efficiency, voltage-electrical current curve, working point, entropy pump mode, generator mode, power factor, figure of merit, Altenkirch-Ioffe model

## Abstract

The basic principles of thermoelectrics rely on the coupling of entropy and electric charge. However, the long-standing dispute of energetics versus entropy has long paralysed the field. Herein, it is shown that treating entropy and electric charge in a symmetric manner enables a simple transport equation to be obtained and the power conversion and its efficiency to be deduced for a single thermoelectric material apart from a device. The material’s performance in both generator mode (thermo-electric) and entropy pump mode (electro-thermal) are discussed on a single voltage-electrical current curve, which is presented in a generalized manner by relating it to the electrically open-circuit voltage and the electrically closed-circuited electrical current. The electrical and thermal power in entropy pump mode are related to the maximum electrical power in generator mode, which depends on the material’s power factor. Particular working points on the material’s voltage-electrical current curve are deduced, namely, the electrical open circuit, electrical short circuit, maximum electrical power, maximum power conversion efficiency, and entropy conductivity inversion. Optimizing a thermoelectric material for different working points is discussed with respect to its figure-of-merit zT and power factor. The importance of the results to state-of-the-art and emerging materials is emphasized.

## 1. Introduction

### 1.1. Controversial Points of View

Entropy is a central quantity in thermoelectrics, but seldom has it been addressed as such. The basic physical quantity that is known today as entropy is widely considered to be a derived quantity according to the approaches by Clausius [1,2,3] and Boltzmann [4,5,6] to quantify its value in certain situations. Both the perception of entropy as a derived quantity and the underestimation of its role in thermal processes are seen as residual outcomes of the Ostwald-Boltzmann battle, which is worth recalling and constitutes another chapter in the tragicomical history of thermodynamics [7]. In the frame of this work, entropy is considered to be a basic quantity. The benefits of this controversial point of view are made obvious on the example of thermoelectric materials.

### 1.2. Implications of Natural Philosophy

Clausius intended to borrow terms for important quantities from the ancient languages, so that they may be adopted unchanged in all modern languages. He proposed to call the quantity *S*, which had been introduced by him, the entropy of the body, from the Greek word τρoπη (tropy), transformation [1,2,3]. Intentionally, he formed the word entropy to be as similar as possible to the word energy. In his opinion, the two quantities to be denoted by these words are so nearly allied in their physical meanings that a certain similarity in designation is desirable [1,2,3].

The importance of entropy was underlined by Gibbs in the very first words of his treatise on thermodynamics: “The comprehension of the laws which govern any material system is greatly facilitated by considering the energy and entropy of the system in the various states of which it is capable” [8,9]. However, the “Energeticist” [10] school in Germany, which rejected atomism and other matter theories, postulated energy as the primary substance in nature, and considered entropy as a superfluous derived concept [11,12,13]. The protagonist was Ostwald, cofounder of physical chemistry and its Nestor in Germany, and behind it was the natural philosophy of Mach [6,14,15]. Soon, the “Energeticist” school attracted much critical attention not only by the British pioneers [16] but also from a younger generation of German physicists [11]. The young Sommerfeld witnessed a memorable debate at the 1895 Assembly of the German Society of Scientists and Physicians in Lübeck, in which Boltzmann “like a bull defeated the torero [Helm as substitute to Ostwald] despite all his art of fencing [14].” In a follow-up critique, Boltzmann [17,18] condemned Ostwald’s “Energetics” not only for perceived mathematical and physical error, but also for its false promise of easy rewards [11]. However, Ostwald never admitted that he had been defeated, and the object of the dispute has been kept alive to the present day [19,20]. Even though the personalities have changed over time, the battle has been newly inflamed in the controversy regarding the Karlsruhe Physics Course [21], which resulted in removing the entropy-treating educational course from German schools [22].

Today, the dissipation or “degradation” of energy is often treated without clear reference to entropy [19,20]. Preference is given to thermal energy (“heat”) or enthalpy. Textbooks on classical thermodynamics take the approach of Clausius to quantify entropy in equilibrium conditions as the definition of entropy, which then is perceived as an energy-derived quantity. The success of Boltzmann’s principle (called so by Einstein [6]) to quantify entropy in partitioned systems in equilibrium [23] renders it often to be a statistics-derived quantity [24]. However, the special cases considered herein do show only certain aspects of entropy, which should be considered in a wider context. By not considering entropy as a central basic quantity, clearness is lost, and uncertainty even creeps over authors who endeavor for accuracy and clarity when it comes to the description of thermal phenomena.

### 1.3. Evolution of Thermodynamics

The field of thermodynamics has evolved from the aim of understanding the thermodynamical engine (i.e., the steam engine) [11], which by principle operates under non-equilibrium conditions. However, for several reasons, thermodynamics has been limited to equilibrium conditions for a long time. For its suggestion to use entropy under non-equilibrium conditions, Planck’s PhD thesis [25] was heavily criticized [19,20]. Planck was likely then intimidated and did not deepen this approach to entropy [19,20]. Alternately, the elegance and success of Gibbs’ treatise on using equilibrium conditions did pave the way for thermodynamics under equilibrium conditions.

It took several decades until Callen [26,27] and de Groot [28] independently formulated a theory to describe thermodynamic systems in non-equilibrium conditions. This theory was helpful for quantitatively describing thermoelectric phenomena. However, the primary focus was the entropy production in irreversible processes and, thus, the excess entropy. No attention was given to entropy itself and its ability, which in older terms could be mentioned as the motive power of entropy, to drive a steam engine [29,30,31] or thermoelectric generator [32,33,34].

### 1.4. Modern Thermodynamics

Consistent with Falk [35], Fuchs [32], and Strunk [23,31], the author holds the view that entropy should be considered as a fundamental quantity. The characteristics of a fundamental quantity unfold from its relations with other fundamental quantities. Concise theories have been developed by Fuchs [32], Job & Rüffler [36,37], and Strunk [23,31,38].

In context of the development of physical concepts, it is worth noting that the basic physical quantity that is known today as entropy, was named quantity of heat by Joseph Black (1728–1799) [39,40,41] and calorique by Sadi Carnot (1796–1832) [29,30,40]. Indeed, calorique is the French word for quantity of heat. In his 1911 Presidential address to the Physical Society of London, Hugh Longbourne Callendar [29] outlined Carnot’s calorique (i.e., entropy) as a quantity, that “any schoolboy could understand”. Moreover, Callendar underlined that Carnot’s calorique reappeared as a triple integral in Kelvin’s 1852 paper, as the thermodynamic function of Rankine and as equivalence-value of a transformation in the 1854 paper of Clausius, and as entropy in the 1865 paper of Clausius [2] along with an abstract redefinition. No one at that time appears to have realized that entropy was merely calorique under another name. Callendar closed his remarks with the advice to distinguish a quantity of heat from a quantity of thermal energy.

Traditionally, thermal energy is called “heat”. Concordant with Callendar [29] and Fuchs [32], in the author’s opinion, heat is not energy, and entropy is the true measure of a quantity of heat as opposed to a quantity of thermal energy. Thus, the use this term for thermal energy should be avoided [42]. For clarity, the traditional term “heat” is put into quotation marks when it addresses the thermal energy. In this approach, entropy is a basic quantity. Thermoelectrics is an example par excellence to show the benefits of this philosophical perspective.

### 1.5. Entropy in Thermoelectrics

In the context of thermoelectrics, according to Boltzmann’s principle, entropy is considered as a statistics-derived quantity when it is used to quantify the effect of spin and orbital degrees of freedom on the Seebeck coefficient in strongly correlated electron systems [43,44]. This, however, is a minor aspect. The approach by Clausius, to consider entropy as an energy-derived quantity does not play a significant role either.

In the so-called theory of thermodynamics of irreversible processes, as developed by Callen [26,27] and de Groot [28], it is rather the case that the thermal energy is derived from the entropy. Entropy is a fundamental quantity that is central to thermoelectrics. These texts can be read with great earning if entropy is considered as an indestructible substance-like quantity that is able to flow through the thermoelectric material and carries the thermal energy. The concept of energy carriers was developed by Falk et al. [45] and Herrmann [21].

However, the theory of thermodynamics of irreversible processes has the tendency to focus on the irreversibly produced excess entropy, but not on the entropy itself. Instead, energetic quantities are preferred. In §60 of his textbook, de Groot [28] presents an alternative presentation of thermoelectricity by the use of entropies of transfer, for which he has stated that the theory becomes somewhat more elegant compared to using energies of transfer. Unfortunately, he has not deepened this approach.

In a preceding paper [34], the author has shown that the rehabilitation of entropy into the theory by Callen [26,27] and de Groot [28] leads to a vivid description of thermoelectric devices. Like electrical charge carries the electrical energy, entropy carries the thermal energy. Thermal induction of an electrical current and electrical induction of a thermal current become understandable.

### 1.6. Aim of This Work

Like the preceding paper by the author [34], the present work aims to contribute to a better understanding of thermoelectrics by reconsidering it by treating entropy and electric charge as basic quantities of equal rank. This is semantically considered by naming the part of energy that flows together with entropy the thermal energy and part of energy flowing together with electrical charge the electrical energy. The energy flux through the thermoelectric material can thus be divided into thermal power and electrical power. Power conversion, which is in the focus of this article, implies that the system under consideration is not in equilibrium, but instead flown through by substance-like quantities. For the case of thermoelectric materials, these are entropy, electric charge, and energy.

By recalling the historical development of the perception of entropy, obstacles are identified, which have hindered the recognition of its important role in the field of thermoelectrics. The confused traditional approach and the use of model devices are avoided. Both power conversion and the efficiency of power conversion are accessed quantitatively for a thermoelectric material apart from a device. New physical insight into thermoelectrics is gained on the level of the thermoelectric material rather than on the device level. On the material’s voltage–electrical current curve, distinct working points are identified (see Table 1), which not only allow for quantification of the material’s properties and performance under specific operational conditions, but also relate generator mode (thermal-to-electrical power conversion) and entropy pump mode (electrical-to-thermal power conversion) of the same material to each other.

The results are worked out in detail, and the outcome from the formalism is graphically illustrated and explained. The simplicity of thermoelectrics is clarified. The findings are linked to the outcome of the traditional approach to thermoelectrics and state-of-the-art thermoelectric materials.

## 2. Results

### 2.1. Categories

The results section is categorized, as follows.

- Section 2.2: Coupling currents of entropy and charge in thermoelectric materials
- Section 2.3: Material’s voltage–electrical current and electrical power–electrical current characteristics
- Section 2.4: Material’s thermal conductivity–electrical current characteristics
- Section 2.5: Thermoelectric material in generator mode
- Section 2.5.1: Working point for maximum electrical power
- Section 2.5.2: Thermal conductivity
- Section 2.5.3: Thermal power
- Section 2.5.4: Power conversion efficiency (thermal to electrical)
- Section 2.5.5: Working points for maximum conversion efficiency and maximum electrical power
- Section 2.6: Thermoelectric material in entropy pump mode
- Section 2.6.1: Power conversion efficiency (electrical to thermal)
- Section 2.6.2: Electrical and thermal power
- Section 2.7: Complete picture

### 2.2. Coupling Currents of Entropy and Charge in Thermoelectric Materials

When a thermoelectric material is simultaneously placed in a gradient of the electrochemical potential $\nabla \tilde{\mu }$ and a gradient of the temperature ∇T, electrical flux density $j_{q}$, and entropy flux density $j_{S}$ are observed [34,46].

(1) $$\left(\begin{array}{c} j_{q}\\ j_{S} \end{array}\right)=\left(\begin{array}{cc} \sigma & \sigma \cdot \alpha \\ \sigma \cdot \alpha & \sigma \cdot \alpha^{2}+\Lambda_{\mathrm{OC}} \end{array}\right)\cdot \left(\begin{array}{c} -\nabla \tilde{\mu }/q\\ -\nabla T \end{array}\right)$$

With the classical thermodynamic potential gradients $\nabla \tilde{\mu }$ (per electric charge *q*) and ∇T being employed, the basic transport Equation (1) has the following structure.

(2) fluxdensities=materialtensor·potentialgradients

The thermoelectric material tensor in Equation (1) is composed of only three quantities, which are the isothermal electrical conductivity σ, the Seebeck coefficient α, and the entropy conductivity at electrical open circuit $\Lambda_{\mathrm{OC}}$ (i.e., at vanishing electrical current). In principle, all three quantities are tensors themselves, but, for homogenous materials, they are often treated as scalars.

The entropy conductivity Λ is related to the traditional “heat” conductivity λ by the absolute temperature *T* [32,34,37]. This, in principle, indicates that the traditional “heat” conduction is based on a more fundamental entropy conduction. The author proposes using the generic term thermal conductivity to address either the “heat” conductivity or the entropy conductivity [47,48].

(3) λ=T·Λ

It is emphasized that Equation (1) refers to a steady-state non-equilibrium situation. Instead of the quantities electric charge *q* and entropy *S*, their local flux densities appear. According to Falk [35], considering local flux densities allows addressing local energy conversion or better to say local power conversion. Because flowing quantities are involved, preference should be given to local power density. Remember, power is the flux of energy. Equation (1) allows for locally varying quantities to be considered, which can be expressed with the positional vector r: $j_{q}=j_{q}\left(r\right)$, $j_{S}=j_{S}\left(r\right)$, $\sigma =\sigma \left(r\right)$, $\alpha =\alpha \left(r\right)$, $\Lambda_{\mathrm{OC}}=\Lambda_{\mathrm{OC}}\left(r\right)$, $\nabla \tilde{\mu }=\nabla \tilde{\mu }\left(r\right)$, $\nabla T=\nabla T\left(r\right)$. Of course, the thermodynamic potentials are locally varying when gradients are present: $\tilde{\mu }=\tilde{\mu }\left(r\right)$, $T=T\left(r\right)$.

However, if the local variation of all quantities in Equation (1) is neglected, a simplified formulation of the transport equation can be observed [34,49,50]. If a further weak temperature dependence is assumed for the electron chemical potential μ (i.e., $\frac{\partial \mu }{\partial T}\approx 0$), the temperature dependence of the electrochemical potential $\tilde{\mu }=\mu +q\cdot \varphi$ is only in the electrical potential φ. With ∇μ/q≈0 follows $\nabla \tilde{\mu }/q=\nabla \mu /q+\nabla \varphi \approx \nabla \varphi$. The assumption of constant gradients (i.e., linear potential curves) allows for them to be substituted by the difference of the respective potential along the thermoelectric material of length *L*: ∇φ→−Δφ/L, ∇T→−ΔT/L. Furthermore, for a thermoelectric material of cross-sectional area *A*, the local flux densities can be replaced by the integrative currents of electrical charge and entropy, respectively: $j_{q}\to I_{q}/A$, $j_{S}\to I_{S}/A$. Subsequently, the transport equation follows as:

(4) $$\left(\begin{array}{c} I_{q}\\ I_{S} \end{array}\right)=\frac{A}{L}\cdot \left(\begin{array}{cc} \sigma & \sigma \cdot \alpha \\ \sigma \cdot \alpha & \sigma \cdot \alpha^{2}+\Lambda_{\mathrm{OC}} \end{array}\right)\cdot \left(\begin{array}{c} \Delta \varphi \\ \Delta T \end{array}\right)$$

**Figure 1:**
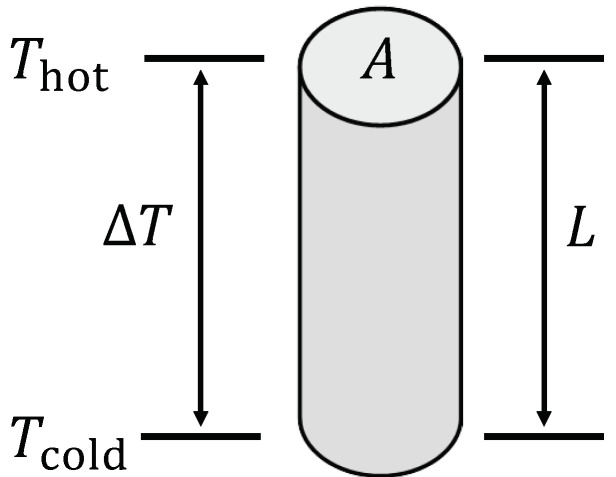
This paper discusses characteristics of a thermoelectric material of cross-sectional area *A* and length *L* when exposed to a temperature difference $\Delta T=T_{\mathrm{hot}}-T_{\mathrm{cold}}$ between a hot reservoir at $T_{\mathrm{hot}}$ and a cold reservoir at $T_{\mathrm{cold}}$.

Equation (4) describes the coupling of currents of electrical charge $I_{q}$ and entropy $I_{S}$ in the thermoelectric material, which causes the occurence of either an electrically-induced entropy current [51] (Peltier effect) or a thermally-induced electrical current [52,53] (Seebeck effect). Note that Equation (4) describes these effects in a thermoelectric material, which is schematically shown in Figure 1, apart from a device.

### 2.3. Material’s Voltage—Electrical Current and Electrical Power—Electrical Current Characteristics

Different working conditions of the thermoelectric material in this article are discussed with reference to the voltage–electrical current curve, which is derived from Equation (4) as Equation (5). Remember that the voltage Δφ is the electrical potential difference along the thermoelectric material.

(5) $$\Delta \varphi =-\alpha \cdot \Delta T+\frac{I_{q}}{\frac{A}{L}\cdot \sigma }$$

According to Equation (5), the voltage–electrical current characteristics is a line, which has the material’s electrical resistance $R=\frac{1}{\frac{A}{L}\cdot \sigma }$ as its slope. This line is only determined by the voltage $\Delta \varphi_{\mathrm{OC}}$ under electrically open-circuited conditions (i.e., at zero electrical current) and the electrical current $I_{\mathrm{SC}}$ at electrically short-circuited conditions (i.e., at zero voltage). The OC is of practical importance for the measurement of temperature using thermocouples.

(6) $$\Delta \varphi_{\mathrm{OC}}=-\alpha \cdot \Delta T$$

(7) $$I_{q,\mathrm{SC}}=\frac{A}{L}\cdot \alpha \cdot \sigma \cdot \Delta T$$

Obviously, the sign of the Seebeck coefficient α determines the sign of both the voltage $\Delta \varphi_{\mathrm{OC}}$ under electrically short-circuited conditions and the electrical current $I_{q,\mathrm{SC}}$ under electrically short-circuited conditions. Thus, the voltage–electrical current characteristics of *p*-type (α>0) or *n*-type (α<0) conductors differ from each other by principle (cf. Appendix A).

**Figure 2:**
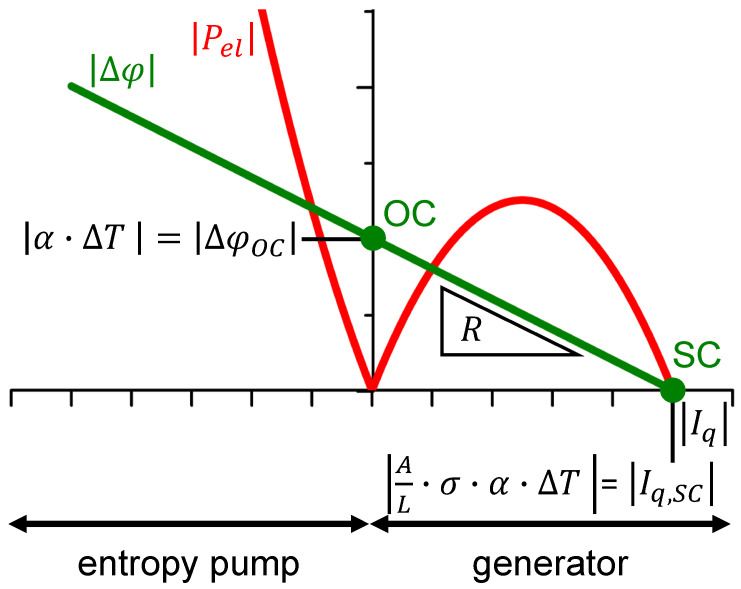
Absolute voltage ∣Δφ∣ – electrical current $|I_{q}|$ curve (green), with slope given by the electrical resistance $R=\frac{1}{\frac{A}{L}\cdot \sigma }$, and the absolute electrical power $|P_{\mathrm{el}}|$ – electrical current $|I_{q}|$ curve (red) for a thermoelectric material. Here, $\Delta T=\frac{T_{\mathrm{hot}}-T_{\mathrm{cold}}}{T_{\mathrm{hot}}}$ is the temperature difference along the thermoelectric material of cross-sectional area *A* and length *L*. These quantities, together with the (isothermal) electrical conductivity σ and the Seebeck coefficient α, determine the electrical current $I_{\mathrm{SC}}$ under electrically short-circuited conditions. The voltage $\Delta \varphi_{\mathrm{OC}}$ under electrically open-circuited conditions is determined by the Seebeck coefficient and the temperature difference. Generator mode refers to a positive sign and entropy pump mode to a negative sign of the electrical power (cf. Appendix A).

To discuss the materials independently of the sign of the Seebeck coefficient, the absolute of the voltage ∣Δφ∣ is plotted in Figure 2 versus the absolute value of the electrical current $|I_{q}|$. In order to diminish Ohmic losses, the electrical resistance $R=\frac{1}{\frac{A}{L}\cdot \sigma }$ must be reduced, which, for the given geometry, requires the electrical conductivity σ to be increased.

To make the discussion independent from even the material parameters and temperature difference ΔT, the normalized electrical current *i* and normalized voltage *u*, as normalized to electrically short-circuited and open-circuited conditions, respectively, are considered in subsequent sections.

(8) $$i=\frac{I_{q}}{I_{q,\mathrm{SC}}}=\frac{I_{q}}{\frac{A}{L}\cdot \alpha \cdot \sigma \cdot \Delta T}$$

(9) $$u=\frac{\Delta \varphi }{\Delta \varphi_{\mathrm{OC}}}=\frac{\Delta \varphi }{-\alpha \cdot \Delta T}=1-i$$

The electrical power $P_{\mathrm{el}}$ is determined by the product of voltage and electrical current as given by Equation (10). It increases linearly with the electrical current, but it is parabolically damped at high electrical currents due to the limited electrical conductivity (Ohmic dissipation [54]).

(10) $$\begin{array}{ccc} P_{\mathrm{el}} & =\Delta \varphi \cdot I_{q} & =\left(-\alpha \cdot \Delta T+\frac{I_{q}}{\frac{A}{L}\cdot \sigma }\right)\cdot I_{q}\\ \; & \; & =-\alpha \cdot \Delta T\cdot I_{q}+\frac{{I_{q}}^{2}}{\frac{A}{L}\cdot \sigma }\\ \; & \; & =-\frac{A}{L}\cdot \sigma \cdot \alpha^{2}\cdot {\left(\Delta T\right)}^{2}\cdot \left(i-i^{2}\right) \end{array}$$

The absolute of the electrical power $|P_{\mathrm{el}}|$ is plotted in Figure 2 versus the absolute value of the electrical current $|I_{q}|$ to discuss the thermoelectric materials independent of the sign of the Seebeck coefficient.

It is obvious from Figure 2 that the electrical power to be put into the material in entropy pump mode may distinctly exceed the electrical power that can be gained in generator mode if the material is applied to the same temperature difference.

### 2.4. Material’s Thermal Conductivity—Electrical Current Characteristics

From Equation (4), the entropy current $I_{S}$ flowing through the material is obtained. It depends on not only the temperature difference ΔT but also the Peltier effect that is associated with the thermally induced electrical current $I_{q}$, which can be expressed by the normalized electrical current *i* as given in Equation (8).

(11) $$\begin{array}{cc} I_{S} & =\frac{A}{L}\cdot \Lambda_{\mathrm{OC}}\cdot \Delta T+\alpha \cdot I_{q}\\ \; & =\frac{A}{L}\cdot \Lambda_{\mathrm{OC}}\cdot \Delta T+\frac{A}{L}\cdot \sigma \alpha^{2}\cdot i\cdot \Delta T\\ \; & =\frac{A}{L}\cdot \left(\Lambda_{\mathrm{OC}}+\sigma \alpha^{2}\cdot i\right)\Delta T\\ \; & =\frac{A}{L}\cdot \Lambda \cdot \Delta T \end{array}$$

From Equation (11), it follows that the thermal conductivity, expressed here by the entropy conductivity Λ, is dependent on the electrical current *i*.

(12) $$\Lambda =\Lambda \left(i\right)=\Lambda_{\mathrm{OC}}+\sigma \alpha^{2}\cdot i$$

When compared to electrically open-circuited conditions, the power factor $\sigma {\dot{\alpha }}^{2}$ gives an additional contribution to the entropy conductivity, which increases linearly with the electrical current. Under electrically short-circuited conditions (SC, i.e., i=1), the entropy conductivity reaches its maximum value.

(13) $$\Lambda_{\mathrm{SC}}=\Lambda_{\mathrm{OC}}+\sigma \alpha^{2}$$

Under electrically short-circuited conditions, the electrical potential is spatially constant (i.e., its gradient vanishes: ∇φ=0). Note that the entropy conductivity at electrical short circuit $\Lambda_{\mathrm{SC}}$, as given by Equation (13), is identical to tensor element $M_{22}$ of the thermoelectric material tensor in the transport Equation (4).

To discuss the characteristics of the entropy conductivity in a general manner, it is normalized to its value under electrically open-circuited conditions:

(14) $$\tilde{\Lambda }=\tilde{\Lambda }\left(i\right)=\frac{\Lambda }{\Lambda_{\mathrm{OC}}}=1+\frac{\sigma \alpha^{2}}{\Lambda_{\mathrm{OC}}}\cdot i=1+zT\cdot i$$

In Equation (14), a figure-of-merit zT has been identified, which only depends on the three material parameters σ, α and $\Lambda_{\mathrm{OC}}$, which make up the material tensor of Equation (4).

(15) $$zT=\frac{\sigma \cdot \alpha^{2}}{\Lambda_{\mathrm{OC}}}$$

**Figure 3:**
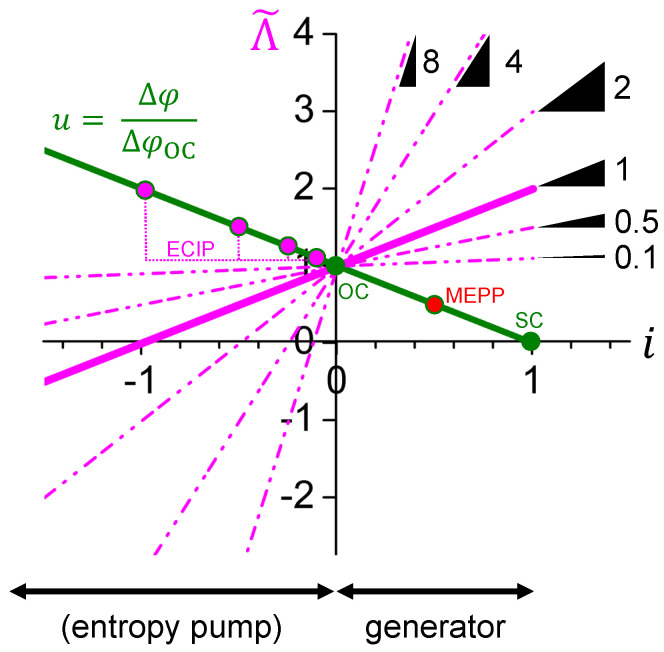
Normalized entropy conductivity $\tilde{\Lambda }$ as function of normalized electrical current *i* for some hypothetical thermoelectric materials. Depending on the figure-of-merit zT, the curves pivot through the working point for electrically open-circuited (OC) conditions. The figure-of-merit zT gives the slope of the curve and its negative reciprocal −1/zT indicates the entropy conductivity inversion point (ECIP). For some thermoelectric materials, the respective ECIP is indicated as working point on the normalized voltage *u*–normalized electrical current *i* curve. Note that the ECIP for materials with zT=0.1. and zT=0.5 is out of the applied scale. The term entropy pump mode is put into brackets because a net entropy current against the temperature difference will only occur if the magnitude of the electrical current is beyond the respective ECIP. For generator mode, the working points MEPP and SC are indicated.

Equation (14) is visualized in Figure 3 for some hypothetical thermoelectric materials with zT=0.1,0.5,1,2,4and8. Working points for electrically open-circuited (OC) conditions, maximum electrical power point (MEPP), and electrical short-circuited (SC) conditions are indicated on the voltage–electrical current curve. Note that the entropy conductivity inversion point (ECIP) is given by the negative reciprocal of the figure-of-merit −1/zT. Only for electrical currents being below the ECIP, effective entropy pump mode is reached with a negative entropy conductivity of the thermoelectric material. Only then, more entropy is pumped against the temperature difference than flows down it. Obviously, the measurements of the thermal conductivity of a thermoelectric material must refer to the working point on the voltage–electrical current curve.

### 2.5. Thermoelectric Material in Generator Mode

#### 2.5.1. Working Point for Maximum Electrical Power

Remember, the characteristics of the thermoelectric material are all discussed for ΔT being different from zero, which implies non-isothermal conditions. It can be easily seen from Equation (10) that maximum electrical power output is obtained for half of the electrically short-circuited electrical current ($i_{\mathrm{MEPP}}=\frac{1}{2}$, cf. Appendix B.1):

(16) $$P_{\mathrm{el},}\;\max=|P_{\mathrm{el}}\left(i_{\mathrm{MEPP}}=0.5\right)|=\frac{1}{4}\cdot \frac{A}{L}\cdot \sigma \cdot \alpha^{2}\cdot {\left(\Delta T\right)}^{2}$$

**Figure 4:**
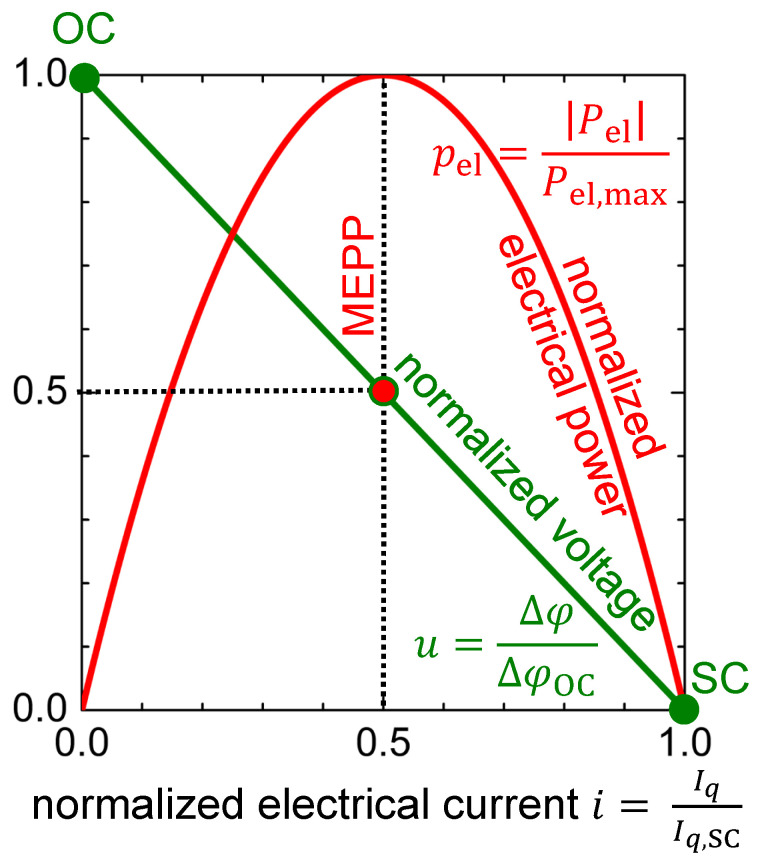
Normalized curves for both voltage *u* – electrical current *i* characteristics and electrical power $p_{\mathrm{el}}$–electrical current *i* characteristics of a thermoelectric material when it is operated in generator mode. The working points open-circuited (OC), maximum electrical power point (MEPP), and short-circuited (SC) are indicated.

To make the discussion independent from material parameters and temperature difference, the normalized electrical power $p_{\mathrm{el}}$, as normalized to the maximum electrical power in generator mode, is plotted in Figure 4.

(17) $$p_{\mathrm{el}}=\frac{|P_{\mathrm{el}}|}{P_{\mathrm{el},}\;\max}=4\cdot |i-i^{2}|$$

The maximum electrical power point (MEPP) is indicated on the normalized voltage–electrical current curve in Figure 4. It is clearly seen that the MEPP ($i_{\mathrm{MEPP}}=0.5$, $u_{\mathrm{MEPP}}=0.5$) is at half of the open-circuited voltage as well as at half of the electrically short-circuited electrical current, which also follows from Equation (9).

#### 2.5.2. Thermal Conductivity

**Figure 5:**
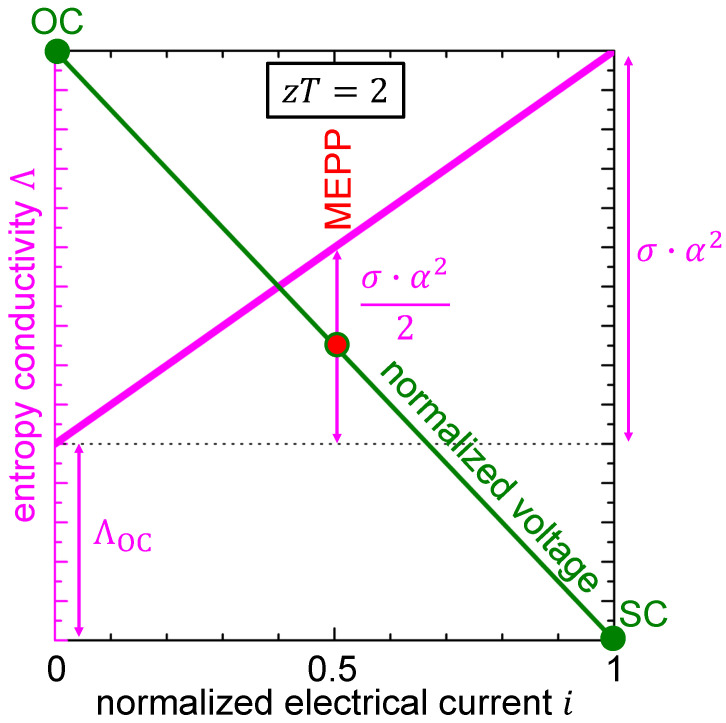
Entropy conductivity Λ as function of the normalized electrical current *i* for a thermoelectric material with zT=2 in generator mode. The working points OC, MEPP, and SC are indicated on the normalized voltage–electrical current curve.

For the thermoelectric material being operated in generator mode, Equation (12) is graphically expressed in Figure 5. The electrically open-circuited entropy conductivity $\Lambda_{\mathrm{OC}}$ is purely dissipative, while the part of the entropy conductivity depending on the power factor $\sigma \cdot \alpha^{2}$ couples to the electrical current, and it fully contributes to the thermal-to-electric power conversion. Obviously, to maximize the electrical power at a given temperature difference, the power $\sigma \cdot \alpha^{2}$ must be maximized, which is in accordance with Equation (10).

The thermally induced electrical current carries electrical energy, which, however, with increasing electrical current, is diminished by Ohmic losses due to the limited (isothermal) electrical conductivity σ as discussed above. At maximum electrical power, the entropy conductivity is increased by half of the power factor as compared to electrically open-circuited conditions. Under electrically short-circuited conditions, the entropy conductivity reaches its maximum (see Equation (13)).

#### 2.5.3. Thermal Power

The thermal input power and the thermal output power depend on the electrical current *i*. According to Fuchs [33], the available thermal power $P_{\mathrm{th}}$ is determined by the fall of entropy down the temperature difference ΔT along the material.

(18) $$P_{\mathrm{th}}=I_{S}\cdot \Delta T=\Lambda \cdot {\left(\Delta T\right)}^{2}=\frac{A}{L}\cdot \left(\Lambda_{\mathrm{OC}}+\sigma \alpha^{2}\cdot i\right)\cdot {\left(\Delta T\right)}^{2}$$

Thus, the available thermal power, as given by Equation (18), depends on the electrical current in the same manner as the entropy conductivity in Figure 3 and Figure 5.

#### 2.5.4. Power Conversion Efficiency (Thermal to Electrical)

From Equations (10) and (18), the second-law power conversion efficiency for the thermoelectric material in generator mode is obtained:

(19) $$\begin{array}{ccc} \eta_{\mathrm{II},\mathrm{gen}} & =|\frac{P_{\mathrm{el}}}{P_{\mathrm{th},\mathrm{avail}}}| & =\frac{\frac{A}{L}\cdot \sigma \cdot \alpha^{2}\cdot {\left(\Delta T\right)}^{2}\cdot \left(i-i^{2}\right)}{\frac{A}{L}\cdot \left(\Lambda_{\mathrm{OC}}+\sigma \alpha^{2}\cdot i\right)\cdot {\left(\Delta T\right)}^{2}}\\ \; & \; & =\frac{i-i^{2}}{i+\frac{\Lambda_{\mathrm{OC}}}{\sigma \cdot \alpha^{2}}}\\ \; & \; & =\frac{i-i^{2}}{i+\frac{1}{zT}} \end{array}$$

**Figure 6:**
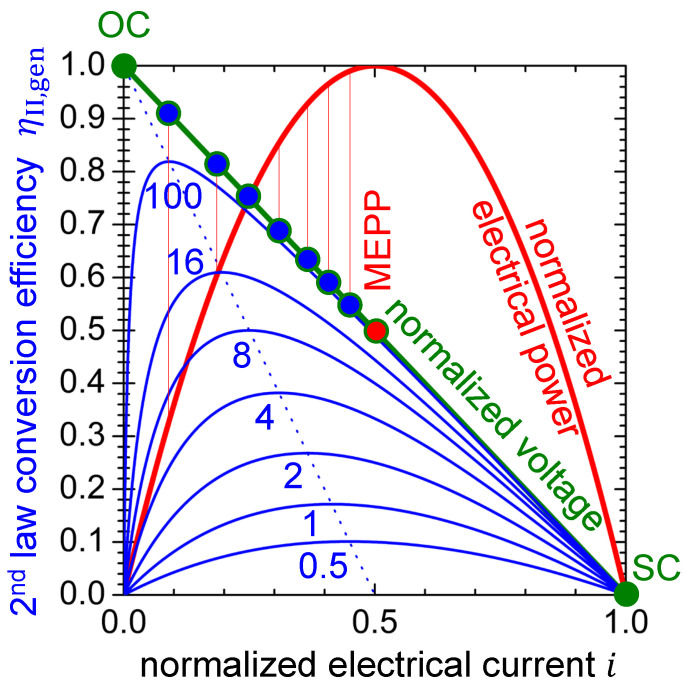
Thermal to electrical power conversion efficiency for some hypothetic materials with figure-of-merit zT varying from 0.5 to 100. Respective working points MCEP (blue) are indicated on the voltage–electrical current curve as well as the MEPP (red). Vertical lines indicate the electrical power output at the MCEP for the example materials. Note that the MCEP drifts apart from the MEPP with increasing figure-of-merit zT. The dashed line indicates the dependence of the MCEP with varying zT.

Equation (19) is plotted in Figure 6 as solid blue curves for some hypothetical thermoelectric materials with different values of the figure-of-merit zT. Obviously, the figure-of-merit zT must be maximized in order to maximize the thermal-to-electrical power conversion efficiency at a given (thermally induced) electrical current.

Equation (19) can be read as the coupled thermal power being converted into electrical power with the constraint; however, with increasing electrical current, Ohmic dissipation gains overhead. As a result, the optimum power conversion efficiency is obtained at lower electrical current than the optimum electrical power output, and the working points for one or other task differ from each other, which can be seen in Figure 6.

According to Fuchs [33], the second-law efficiency $\eta_{\mathrm{II},\mathrm{gen}}$ is related to the first-law efficiency $\eta_{I,\mathrm{gen}}$ by Carnot’s efficiency $\eta_{C}$.

(20) $$\eta_{I,\mathrm{gen}}=\eta_{C}\cdot \eta_{\mathrm{II},\mathrm{gen}}=\frac{T_{\mathrm{hot}}-T_{\mathrm{cold}}}{T_{\mathrm{hot}}}\cdot \eta_{\mathrm{II},\mathrm{gen}}$$

Carnot’s efficiency $\eta_{C}$ places a theoretical limit for the case in which the second-law efficiency $\eta_{\mathrm{II},\mathrm{gen}}=1$, which refers to the unrealistic case of vanishing dissipation. Nevertheless, the second-law efficiency $\eta_{\mathrm{II},\mathrm{gen}}$ is the only material-dependent factor and has been used by Altenkirch [55] and Ioffe [56] in order to estimate the performance of thermoelectric materials by treating thermogenerators. It is worth noting that the entropy-based approach presented here allows for power conversion and its efficiency for a single thermoelectric material apart from a device to be discussed.

#### 2.5.5. Working Points for Maximum Conversion Efficiency and Maximum Electrical Power

From the maximum of Equation (19), the maximum conversion efficiency point (MCEP) is obtained with the normalized electrical current $i_{\mathrm{MCEP},\mathrm{gen}}$ being, as follows (cf. Appendix B.2):

(21) $$\begin{array}{cc} i_{\mathrm{MCEP},\mathrm{gen}} & =\frac{1}{\sqrt{1+zT}+1} \end{array}$$

At the MCEP, the maximum power conversion efficiency of the thermoelectric material in generator mode is then obtained, as follows (cf. Appendix B.2):

(22) $$\begin{array}{ccc} \eta_{\mathrm{II},\mathrm{gen},\max} & =\eta_{\mathrm{II},\mathrm{gen}}\left(i_{\mathrm{MCEP},\mathrm{gen}}\right) & =\frac{\sqrt{1+zT}-1}{\sqrt{1+zT}+1} \end{array}$$

Equation (23), which shows the variation of the MCEP with varying $i_{\mathrm{MCEP},\mathrm{gen}}$ due to varying zT, is plotted in Figure 6 as dotted blue line.

(23) $$\begin{array}{cc} \eta_{\mathrm{II},\mathrm{gen},\max}\left(i_{\mathrm{MCEP},\mathrm{gen}}\right) & =1-2\cdot i_{\mathrm{MCEP},\mathrm{gen}} \end{array}$$

Note that with increasing figure-of-merit zT, not only does the MCEP drift apart from the MEPP, but the electrical power output also decreases with respect to the MEPP (see Equation (16)), both of which can be seen in Figure 6 (cf. Appendix B.2).

(24) $$\begin{array}{cc} P_{\mathrm{el},\mathrm{MCEP}} & =\frac{4\cdot \sqrt{1+zT}}{{\left(\sqrt{1+zT}+1\right)}^{2}}\cdot P_{\mathrm{el},\max} \end{array}$$

**Figure 7:**
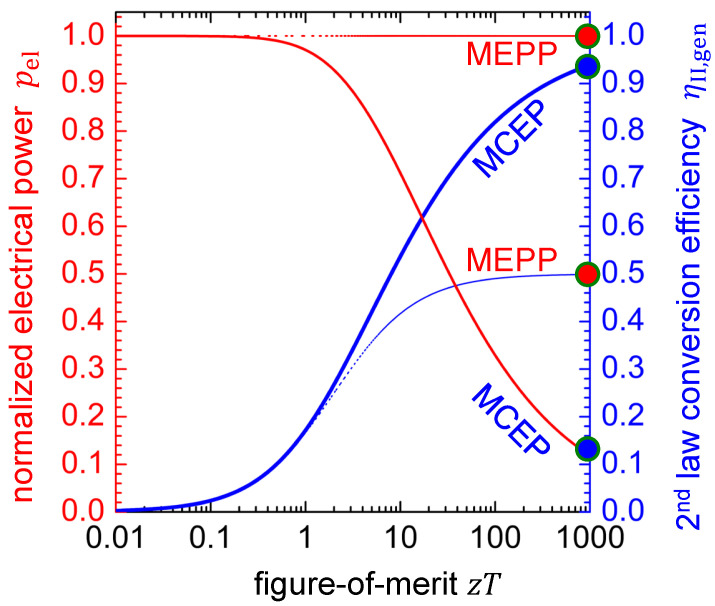
Electrical power output (red lines) and thermal-to-electrical power conversion efficiency (blue lines) for some hypothetic materials with figure-of-merit zT varying from 0.01 to 1000 when operated in two distinct working points, respectively. Solid lines refer to the MCEP and dashed lines refer to the MEPP.

Obviously, with increasing figure-of-merit zT, the electrical power at the MCEP converges to zero. Figure 7 shows that a notable difference in electrical power output between MCEP and MEPP can be expected for thermoelectric materials with zT>0.3 only (red curves). A notable difference in the power conversion efficiency of the thermoelectric material being operated in the MCEP or the MEPP can only be expected when zT>2. This is also obvious from Table 2, which, for some hypothetical values of the material’s figure-of-merit zT, gives values of the second-law power conversion efficiency at the working points under discussion. The 2^nd^ law power conversion efficiency at the MEPP is obtained as follows (cf. Appendix B.1).

(25) $$\begin{array}{ccc} \eta_{\mathrm{II},\mathrm{gen},\mathrm{MEPP}} & =\eta_{\mathrm{II},\mathrm{gen}}\left(i_{\mathrm{MEPP}}=0.5\right) & =\frac{1}{2}\cdot \frac{zT}{zT+2} \end{array}$$

It is worth noting that, for a thermoelectric material with zT<2, there is no benefit from operating it apart from the MEPP.

### 2.6. Thermoelectric Material in Entropy Pump Mode

#### 2.6.1. Power Conversion Efficiency (Electrical to Thermal)

Traditional approaches consider a coefficient of performance when addressing the performance of a thermoelectric cooling or heating device [56,57]. Analogously, a coefficient of performance COP of the thermoelectric material, when used in a cooler, can be considered. It is the thermal power removed from the cold side $T_{\mathrm{cold}}\cdot I_{S}$ related to the electrical power (cf. Appendix C.1).

(26) $$\begin{array}{ccc} {COP}_{\mathrm{cooler}} & =|\frac{T_{\mathrm{cold}}\cdot I_{S}}{P_{\mathrm{el}}}| & =\frac{T_{\mathrm{cold}}}{\Delta T}\cdot |\frac{P_{\mathrm{th}}}{P_{\mathrm{el}}}|\\ \; & \; & =\frac{T_{\mathrm{cold}}}{\Delta T}\cdot \eta_{\mathrm{II},\mathrm{ep}} \end{array}$$

If instead of a cooler, the thermoelectric material is used in a heater (see Fuchs [32], p. 135ff), the thermal power released to the hot side $T_{\mathrm{hot}}\cdot I_{S}$ becomes the reference parameter, and the COP is then (cf. Appendix C.1):

(27) $$\begin{array}{ccc} {COP}_{\mathrm{heater}} & =|\frac{T_{\mathrm{hot}}\cdot I_{S}}{P_{\mathrm{el}}}| & =\frac{T_{\mathrm{hot}}}{\Delta T}\cdot |\frac{P_{\mathrm{th}}}{P_{\mathrm{el}}}|\\ \; & \; & =\frac{T_{\mathrm{hot}}}{\Delta T}\cdot \eta_{\mathrm{II},\mathrm{ep}}\\ \; & \; & =\frac{1}{\eta_{C}}\cdot \eta_{\mathrm{II},\mathrm{ep}} \end{array}$$

In both cases, Equations (26) and (27), the COP can be factorized into a temperature factor and the second-law efficiency for the thermoelectric material in entropy pump mode $\eta_{\mathrm{II},\mathrm{ep}}$ (see Fuchs [32], p. 135ff). When the thermoelectric material is used in a heater (Equation (27)), the temperature factor is the inverse of Carnot’s efficiency $\eta_{C}$ [32]. The second-law efficiency for the thermoelectric material in entropy pump mode $\eta_{\mathrm{II},\mathrm{ep}}$ relates the thermal power $P_{\mathrm{th}}$ that is needed to pump a certain entropy current from the cold side to the hot side to the electrical power $P_{\mathrm{el}}$ (cf. Appendix C.1).

(28) $$\begin{array}{ccc} \eta_{\mathrm{II},\mathrm{ep}} & =|\frac{P_{\mathrm{th}}}{P_{\mathrm{el}}}| & =\frac{i+\frac{1}{zT}}{-i^{2}+i} \end{array}$$

The second-law efficiency for the thermoelectric material in entropy pump mode $\eta_{\mathrm{II},\mathrm{ep}}$ only depends on the normalized electrical current *i* (i.e., working point on the voltage–electrical current curve) and the material’s figure-of-merit zT. It can be used to assess the performance of the thermoelectric material when it is used to pump entropy, regardless of whether the purpose is cooling or heating.

**Figure 8:**
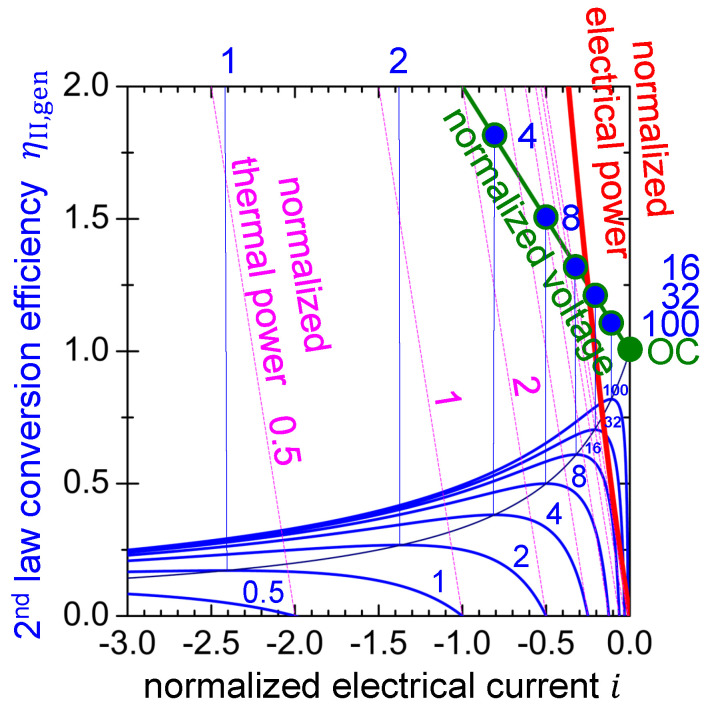
Electrical-to-thermal power conversion efficiency as a function of the reduced electrical current for some hypothetic materials with figure-of-merit zT varying from 0.5 to 100. Respective working points MCEP (blue) are indicated on the voltage–electrical current curve for zT=100,32,18,8and4. Further vertical lines (blue) indicate the MCEP for zT=2,1. The MCEP for zT=0.5 is out of display. The hyperbolic curve indicates the dependence of the MCEP with varying zT. The red curve indicates electrical power–electrical current characteristics. The set of inclined parallel lines (magenta) indicate the thermal power–electrical current characteristics for the respective zT. All of the power curves are normalized to the MEPP in generator mode.

Note that the second-law efficiency for the thermoelectric material in entropy pump mode $\eta_{\mathrm{II},\mathrm{ep}}$ (Equation (28)) is the inverse of the second-law efficiency for the thermoelectric material in generator mode (Equation (19)). Because a net entropy current from the cold side to the hot side will only be obtained for negative entropy conductivity (see Equation (14) and Figure 3), here $\eta_{\mathrm{II},\mathrm{ep}}$ will make sense only for the normalized electrical current being $i\leq \frac{1}{zT}$. For this parameter range it is plotted in Figure 8 for some hypothetic thermoelectric materials with figure-of-merit zT between 0.5 and 100.

The maximum 2^nd^-law power conversion efficiency for a thermoelectric material operated in entropy pump mode is dependent on the material’s figure-of-merit zT (cf. Appendix C.2):

(29) $$\begin{array}{ccc} \eta_{\mathrm{II},\mathrm{ep},\max} & \; & =\frac{\sqrt{1+zT}-1}{\sqrt{1+zT}+1} \end{array}$$

It is obtained at a normalized electrical current $i_{\mathrm{MCEP},\mathrm{ep}}$, which corresponds to the thermoelectric material’s maximum conversion efficiency point (MCEP) in entropy pump mode (cf. Appendix C.2). Respective working points for some hypothetic thermoelectric materials are indicated on the voltage–electrical current curve presented in Figure 8.

(30) $$\begin{array}{cc} i_{\mathrm{MCEP},\mathrm{ep}} & =-\frac{1}{\sqrt{1+zT}-1} \end{array}$$

The dependence of the maximum second-law efficiency on the electrical current is shown in Figure 8 as a hyperbolic line (cf. Appendix C.2).

(31) $$\begin{array}{cc} \eta_{\mathrm{II},\mathrm{ep},\max}\left(i_{\mathrm{MCEP},\mathrm{ep}}\right) & =\frac{1}{1-2\cdot i_{\mathrm{MCEP},\mathrm{ep}}} \end{array}$$

Obviously, an ideal thermoelectric material would have an infinite zT, but the MCEP converges then to the OC working point at vanishing electrical current and, thus, zero electrical power. On the contrary, for the limit of vanishing zT, the maximum second-law efficiency converges to zero at infinite magnitude of the electrical current.

#### 2.6.2. Electrical and Thermal Power

All of the power curves in Figure 8, for the thermoelectric material in entropy pump mode, are normalized to the MEPP in generator mode (see Figure 2 and Figure 4) when the material is exposed to the same temperature difference ΔT. According to Equations (16) and (18), the normalized thermal power $p_{\mathrm{th}}$ in Figure 8 is given by a straight line that intersects the horizontal axis at $-\frac{1}{zT}$ and it has a slope of −4 (cf. Appendix C.3).

(32) $$\begin{array}{ccc} p_{\mathrm{th}} & =\frac{|P_{\mathrm{th}}|}{P_{\mathrm{el},\max}} & =4\cdot |\frac{1}{zT}+i| \end{array}$$

For different values of the figure-of-merit zT, a set of inclined parallel lines results. Only the lines for zT=0.5,1and2 are labelled in Figure 8. With increasing figure-of-merit zT, the normalized thermal power curve approaches the normalized electrical power curve, which is in accordance with the increasing power conversion efficiency. However, when the thermoelectric material is operated in its MCEP, the thermal power will decrease with increasing figure-of-merit zT, which becomes obvious when Equation (30) is combined with Equation (32) (cf. Appendix C.2).

(33) $$\begin{array}{ccc} p_{\mathrm{th},\mathrm{MCEP}} & =p_{\mathrm{th}}\left(i_{\mathrm{MCEP},\mathrm{ep}}\right) & =4\cdot \frac{\sqrt{1+zT}}{zT} \end{array}$$

The normalized thermal power at MCEP would be steeply curved in Figure 8, with the data point out of scale for zT<8, but has been skipped for clarity. Instead, relevant values for the MCEP are listed in Table 3, together with the normalized electrical power and the normalized electrical current.

### 2.7. Complete Picture

**Figure 9:**
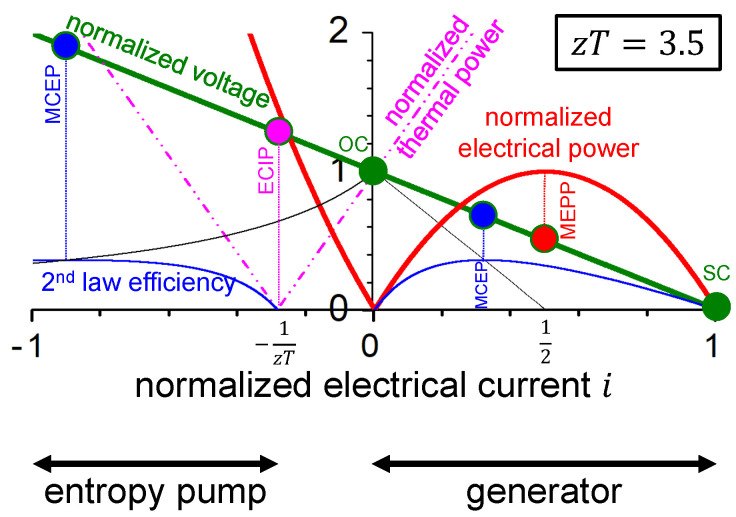
Related characteristics of a hypothetic thermoelectric material with figure-of-merit zT=3.5 in entropy pump mode and generator mode: normalized voltage, normalized electrical power, normalized thermal power, and 2^nd^-law conversion efficiency as a function of the normalized electrical current. Different working points are indicated on the voltage–electrical current curve. Note that, for current state-of-the-art materials, the MCEP in entropy pump mode would be out of display (see Table 3).

With the approach chosen here, working points on the voltage–electrical current curve relate the power conversion properties of the thermoelectric material in generator mode and entropy pump mode to each other. Figure 9 illustrates the concise result for a hypothetical thermoelectric material with figure-of-merit zT=3.5.

For a given figure-of-merit zT, according to Equations (22) and (29), the values of the maximum 2^nd^-law conversion efficiency for both modes are identical. Some values are given in Table 2. In addition, values of the 2^nd^-law conversion efficiency at the MEPP in generator mode are given (see Equation (25)). Remember, the obtained power requires consideration of the absolute value of the electrical power, as determined by the power factor (see Equation (16)).

## 3. Materials and Methods

Detailed calculations, as given in Appendix B and Appendix C, were made using pencil and paper. The manuscript was prepared using Latex in MikTex distribution. Figures were drawn with the aid of Microcal’s Origin and Microsoft’s PowerPoint.

## 4. Discussion

### 4.1. Remarks on the Use of Working Points

Traditionally, a thermoelectric device is considered and, in generator mode, the operational conditions are set by an external load resistance. The approach of this work, which uses working points on the material’s voltage–electrical voltage curve, gives consistent results, which is explicitly shown in Appendix B.3. However, consideration of working points comes with the advantage that the contribution of individual thermoelectric materials in a device can be easily understood [58]. Moreover, the material’s voltage–electrical voltage curve directly relates generator mode and entropy pump mode.

### 4.2. Remarks on the Altenkirch-Ioffe Model

**Figure 10:**
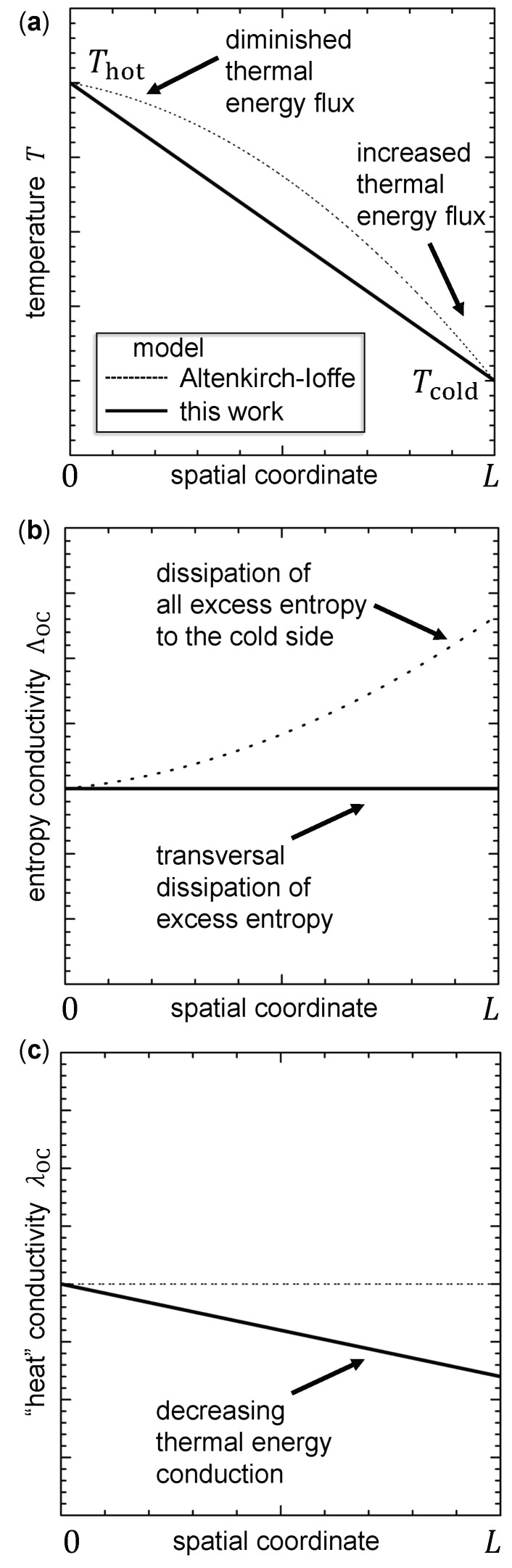
Comparison of the model of this work (constant entropy conductivity) to the Altenkirch-Ioffe model [33,55,56,60] (constant “heat” conductivity) with the schematic profiles of the following quantities over the thermoelectric material when the material is carrying a (thermally induced) electrical current: (**a**) temperature *T*; (**b**) electrically open-circuited entropy conductivity $\Lambda_{\mathrm{OC}}$; and, (**c**) electrically open-circuited “heat” conductivity $\lambda_{\mathrm{OC}}$. Note that profiles are not drawn to scale.

Due to the prominence of the Altenkirch-Ioffe model [55,56], it is worth comparing it to the model, which has been introduced in this work. A comparison of important quantities described by the model of this work and the Altenkirch-Ioffe model is shown in Figure 10.

Remember, Equation (4) has been derived for a thermoelectric material apart from a device. Furthermore, a constant temperature gradient has been assumed, which means a constant slope of the temperature profile, which then connects the hot side at $T_{\mathrm{hot}}$ and the cold side at $T_{\mathrm{cold}}$ by a straight line (solid line in Figure 10a). The further assumption of a temperature-independent entropy conductivity $\Lambda_{\mathrm{OC}}$ at electrical open-circuit is plotted in Figure 10b as a solid line. As a consequence of these assumptions, at a given electrical current (including electrically open-circuited conditions), the entropy current will carry the highest energy current at the hot side of the thermoelectric material. When advancing through the thermoelectric material to lower temperatures, the entropy current cannot further carry all thermal energy (“heat”), which then needs to be dissipated. Following Walstrom’s approach [59], thermal energy is assumed to be dissipated transversally together with instantaneously produced excess entropy as its carrier. It is important to emphasize that excess entropy leaves the thermoelectric material in directions transversal to the flow of the entropy inserted at the hot side. The ability to conduct thermal energy is decreased with decreasing temperature, which is reflected in a decreasing “heat” conductivity, as plotted in Figure 10c as a solid line.

Traced back to Altenkirch [55] and Ioffe [56], often a model is discussed that considers a two-leg thermogenerator and assumes constant “heat” conductivity. Concerning the thermoelectric material, the model is purely one-dimensional and does not allow for transversal dissipation of entropy and energy. All dissipation has to be considered parallel or antiparallel to the flow of entropy and thermal energy along the thermoelectric material. In fact, only the parallel option (i.e., down the temperature gradient) remains physically meaningful. Under electrically open-circuited conditions (i.e., vanishing electrical current), the temperature profile can still be linear. However, Heikes and Ure [60] have shown that, in the presence of a thermally-induced electrical current, the temperature profile is flattened at the hot side and steeply sloping at the cold side, which is shown in Figure 10a as a dashed line. As a consequence of the curved temperature profile and the constant “heat” conductivity (see dashed line in Figure 10c), the “heat” flux is diminished at the hot side (thermal energy input) and increased at the cold side (thermal energy output). The change in the temperature profile is such that, as compared to the zero electrical current situation, the thermal energy input is diminished by half of the Joule “heat” and the thermal energy release at the cold side is increased by half of the Joule “heat”, as shown by Heikes and Ure [60,61]. This is to account for the dissipation of thermal energy being parallel to the flow of entropy and thermal energy. As a consequence, when compared to electrically open-circuited conditions, the thermoelectric material would be thermally less transparent when an electrical current flows.

In contrast, the model of this work predicts the thermoelectric material to become thermally more transparent with increasing electrical current, which is reflected in the then reversible increased entropy conductivity Λ(i) (see Equations (12) and (14)). In the author’s opinion, this is an important characteristic of thermoelectric materials, which is fully embezzled in the traditional model.

In the Altenkirch-Ioffe model, all the excess entropy and excess thermal energy are dissipated to the cold side, which is reflected in an irreversible increase of the entropy conductivity along the thermoelectric material, as visualized in Figure 10b. The aforementioned assumption introduces a ratio of $T_{\mathrm{hot}}/T_{\mathrm{cold}}$ into the formula for the 2^nd^-law efficiency at the MCEP (see Appendix B.4 and Appendix B.5 for a device in generator mode; see Appendix C.4 and Appendix C.5 for a device in entropy pump mode). Ioffe [56] has shown that the deviation from Equation (22) (generator mode) or Equation (29) (entropy pump mode), however, is only a few per cent when the efficiency itself is small. In other words, for a small temperature difference ΔT, both of the models give nearly the same results.

It must be emphasized that both of the models rely on very special assumptions and, thus, cannot claim general validity [62]. In this sense, all of the results have to be considered semi-quantitatively when it comes to real thermoelectric materials and devices. More general considerations, as provided by Equation (1), need to consider the local variation of thermoelectric parameters but are beyond the scope of this work. Heikes and Ure [60] and Gryasnov et al. [63] have considered the local variation of thermoelectric parameters to some extent. However, the advantage of the model of this work is not only to consider the thermoelectric material apart from a device, but also to clearly separate the dissipation of entropy and thermal energy from the reversible thermoelectric coupling. The simplicity of thermoelectrics is manifested.

### 4.3. Remarks on Narducci’s Model

Narducci has put the question “Do we really need high thermoelectric figures of merit?” and found in his calculations that, when considering constant ΔT, the electrical power output of a two-leg thermogenerator device at the MEPP increases with increased thermal conductivity (see Narducci [64], Figure 2). The situation that is discussed by Narducci corresponds to a decreasing figure-of-merit (i.e., zT→0 limit) with the electrical power converging to what we have obtained here as $P_{\mathrm{el},\;\max}$ (see Equation (16)). In light of this work, it becomes obvious that the MCEP and the MEPP of the thermoelectric material(s) then merge (see Figure 6 and Figure 7).

### 4.4. Remarks on $\Lambda_{\mathrm{OC}}$

In the model applied in this work, the electrically open-circuited entropy conductivity $\Lambda_{\mathrm{OC}}$ originates only from non-charge transporting excitations of the solid (mostly phonons). Here, the contribution from electrons to the entropy conductivity solely originates from the power factor (see Figure 3 and Figure 5). Subsequently, distinguishing contributions from electrons and phonons to the thermal conductivity is straightforward (see Ioffe [56], p. 44) and has been coined the "phonon glass–electron crystal" (PGEC) concept by Slack [65]. In this case, $\Lambda_{\mathrm{OC}}$ is identical to the phonon contribution to the entropy conductivity.

However, as mentioned by Ioffe (see Ioffe [56], p. 46), in materials with charge carriers of both signs (electrons and holes from multiple bands), the situation is more intricate. Subsequently, important electronic contribution to the thermal conductivity can be expected for vanishing net flux of charge. In other words, the electrically open-circuited entropy conductivity $\Lambda_{\mathrm{OC}}$ has contributions from both phonons and electrons. The application of the empirical Wiedemann-Franz law to describe the relationship between thermal and electrical conductivity is questionable for these materials [48,56]. In practice, this is the case for many semiconductors and metals. To improve the thermoelectric properties of these materials, it is not sufficient to reduce the phonon contribution by the PGEC concept. In addition, electronic band engineering is required in order to diminish the electron contribution to $\Lambda_{\mathrm{OC}}$. The theory in this work can be easily extended to treat this case by introducing a second type of charge carrier into Equations (1) and (4).

### 4.5. Remarks on Figure-of-Merit zT

In this work, the figure-of-merit has been introduced in context with the entropy conductivity (cf. Equation (14)) to underline that it is the dimensionless ratio of two entropy conductivities. Initially, the thermoelectric figure-of-merit was introduced by Ioffe [56] as a parameter $z=\frac{\sigma \cdot \alpha^{2}}{\lambda_{\mathrm{OC}}}$ in the treatment of a thermogenerator referring to the “heat” conductivity. In subsequent treatment, Ioffe has taken into account the medium temperature $\bar{T}$ of the device and elucidated the thermoelectric material’s figure-of-merit to be $z\bar{T}=\frac{\sigma \cdot \alpha^{2}}{\lambda_{\mathrm{OC}}}\cdot \bar{T}$, which has subsequently been widely used as zT. With this formulation of the figure-of-merit, researchers often have been confused by the intensive variable temperature $\bar{T}$ showing up explicitly besides material parameters [66]. It is seen as a persistent residual outcome of the historical dispute between Ostwald and Boltzmann (see Section 1.2) that it has not been realized that the use of entropy conductivity Λ instead of the “heat” conductivity λ makes the figure-of merit depend on three material parameters only, which all implicitly depend on temperature (see Equations (3) and (15)).

The author has used zT to be consistent with the conventional nomenclature of the thermoelectric community. All of the formulas in this article, which contain the figure-of-merit, however, would look more straightforward if zT were to be substituted by a single letter, for instance, *f* as used by Zener [67].

(34) $$f=\frac{\sigma \cdot \alpha^{2}}{\Lambda_{\mathrm{OC}}}=\frac{\sigma \cdot \alpha^{2}}{\lambda_{\mathrm{OC}}}\cdot T=zT$$

### 4.6. Remarks on State-of-the-Art and Emerging Thermoelectric Materials

It is worth noting that, for a thermoelectric material with zT<2, there is no benefit from operating it apart from the MEPP (see Figure 6, Figure 7 and Table 2). In this context, it is important to perceive that current state-of-the-art materials hardly exceed a zT value of 2. The values listed in Table 4 are peak values. Among the materials of Table 4, PbTe${}_{0.7}$S${}_{0.3}$-2.5%K has a peak zT of 2.2 at 923 K and a record high average zT of 1.56 in the temperature interval of 300–900 K [68]. Conclusively, the tracking of the MEPP [69], but not of the MCEP, is reported for thermogenerators. However, for the application of emerging thermoelectric materials with further improved figure-of-merit, and thus more distant working points, tracking of the MCEP might become relevant.

The benefit of an increased figure-of-merit zT will be an increased power conversion efficiency at the MEPP anyway. Figure 6, Figure 7, and Table 2 indicate that the material’s second-law power conversion efficiency at the MEPP will not exceed the value of 0.5 (see also Equation (25)). Interestingly, this value corresponds to the lower limit of the Curzon-Ahlborn efficiency of a Carnot engine operated at its MEPP [91,92]. At the MEPP, a real thermoelectric material will always be operated at less than half of the Carnot efficiency.

### 4.7. Remarks on the Importance of the Power Factor and Choice of Materials for Thermogenerators

Because normalized curves are discussed in this work, one might lose sight of the fact that the power factor $\sigma \cdot \alpha^{2}$ is at least as important as the figure-of-merit zT. According to Equation (16), it rules over the maximum achievable absolute electrical power when the thermoelectric material is operated in generator mode at MCEP. For a material with high zT (e.g., 100), the electrical power is much lower at the MCEP compared to the MEPP (Figure 6 and Figure 7). This is because, at the low electrical current of the MCEP, the thermoelectric material is less permeable to entropy when compared to the MEPP (see Figure 5). Thus, less thermal power is available to be thermoelectrically converted into electrical power. The amount of useful thermal power depends on the power factor and the electrical current (see the second summand in Equation (18)).

The open-circuited entropy conductivity $\Lambda_{\mathrm{OC}}$ causes a thermoelectrically-inactive bypass, which eventually leads the temperature difference ΔT, which squared determines the maximum electrical power in Equation (16), to drop. To provide large ΔT, the open-circuited entropy conductivity $\Lambda_{\mathrm{OC}}$ should be kept small. Here, in addition to a high power factor $\sigma {\dot{\alpha }}^{2}$, the figure-of-merit zT comes into play, which relates the aforementioned contributions to the entropy conductivity (see Equation (12) and Equation (15)). The materials that are listed in Table 4 represent those with the highest values of the figure-of-merit reported thus far. In the author’s opinion, the most interesting materials are those that also have a high power factor of at least 30 μWcm${}^{-1}$K${}^{-2}$.

A high electrical conductivity σ is also advantageous, as already mentioned in Section 2.3. The choice of materials can easily be made with the help of type-1 Ioffe plots [56] ($\sigma \alpha^{2}-\sigma$) and type-2 Ioffe plots ($\Lambda_{\mathrm{OC}}-\sigma$) [56,93], which have been recently revitalized on the example of current thermoelectric materials [47,48,94]. The reader is referred to Fuchs [32] (p. 135ff) for further details.

### 4.8. Remarks on the Second-Law Power Conversion Efficiency vs. Coefficient of Performance for Entropy Pumps

While the upper limit of the coefficient of performance will depend on temperature conditions, as involved in the Carnot efficiency $\eta_{C}$ (Equation (27)) or the temperature factor $\frac{T_{\mathrm{cold}}}{\Delta T}$ (Equation (26)), the upper limit for the second-law efficiency is fixed to unity (i.e., $\eta_{\mathrm{II},\mathrm{ep}}\leq 1$). The unity value of the second-law efficiency refers to an ideal material. While the coefficient of performance is related to a floating scale, the second-law efficiency allows for the estimation of how far from ideal a thermoelectric material is. Another advantage is that the second-law efficiency in Equation (28) only depends on the figure-of-merit and the electrical current and, thus, allows for evaluation of the performance of the thermoelectric material apart from specific temperature conditions, as well as independent from use in a cooler or a heater.

Note that, according to Equations (29) and (22), the maximum second-law efficiency of a thermoelectric material is identical in entropy pump mode and generator mode:

(35) $$\begin{array}{cccc} \eta_{\mathrm{II},\mathrm{ep},\max} & =\eta_{\mathrm{II},\mathrm{ep}}\left(i_{\mathrm{MCEP},\mathrm{ep}}\right) & =\eta_{\mathrm{II},\mathrm{gen}}\left(i_{\mathrm{MCEP},\mathrm{gen}}\right) & =\eta_{\mathrm{II},\mathrm{gen},\max} \end{array}$$

This is also apparent from Figure 9.

### 4.9. Remarks on the Choice of Materials for Entropy Pumps

Remember, electrical and thermal power in Figure 8 are normalized to the MEPP in generator mode (see Equations (17) and (32)). Thus, the absolute thermal power in entropy pump mode is determined by the material’s power factor $\sigma \cdot \alpha^{2}$ (see Equation (16)). A low open-circuited entropy conductivity $\Lambda_{OC}$ is desired to prevent the thermoelectrically inactive fall of entropy along the temperature difference ΔT, which would make it difficult to maintain the ΔT. Thus, in addition to a high power factor $\sigma \cdot \alpha^{2}$, a high figure-of-merit zT is favourable, which relates the aforementioned quantities (see Equation (15)).

Operating the thermoelectric material in entropy pump mode requires good performance at ambient temperature and below (e.g., for cooling 150–300 K) or above (e.g., for heating 300–400 K). Among the materials listed in Table 4, only bismuth telluride-based materials fulfil all requirements; and, they are the current materials of choice for the mentioned applications and are conclusively found in commercial devices.

According to Figure 8, emerging materials with improved figure-of-merit at a power factor comparable to bismuth telluride-based materials would have the benefit that comparable thermal power could be pumped from the cold to hot side at a lower electrical current and electrical power.

## 5. Conclusions

Treating entropy and electrical charge as basic quantities allows for a concise description of thermoelectric transport phenomena (entropy, charge, thermal energy, and electrical energy) and it is the key to comprehensibility. The basic transport equation involves classical thermodynamic potentials (temperature and electrical potential) and enables the identification of a thermoelectric material tensor. On the material’s voltage–electrical current cure, distinct working points can be identified, which allow for consideration of the power conversion and its efficiency of the thermoelectric material apart from a device. The power depends on the power factor, and the conversion efficiency depends on the figure-of-merit zT. A clear physical meaning is given to the power factor as the part of the entropy conductivity that couples to the electrical current. The thermal conductivity, expressed here as entropy conductivity, depends on the electrical current and becomes negative when the thermoelectric material is operated in entropy pump mode. The dimensionless figure-of-merit zT is the ratio of two entropy conductivities, the one under electrically open-circuited conditions and the one that couples to the electrical current. The performance of the thermoelectric material in generator mode and entropy pump mode are related to each other and they can be considered on a single voltage–electrical current curve apart from a device.

## Figures and Tables

**Table 1:** Working points on the voltage–electrical current curve of a thermoelectric material in both operational modes, as addressed in this work.

Abbreviation	Working Point	Operational Mode
MCEP	Maximum (power) conversion efficiency point	entropy pump mode
EICP	Entropy conductivity inversion point	entropy pump mode
OC	(electrical) open circuit	generator mode
MCEP	(see above)	generator mode
MEPP	Maximum (electrical) power point	generator mode
SC	(electrical) short circuit	generator mode

**Table 2:** Second-law power conversion efficiency of a thermoelectric material at the MCEP in either entropy pump mode or generator mode and at the MEPP in generator mode for some hypothetical values of the figure-of-merit zT.

zT	Maximum 2^nd^ Law Efficiency	2^nd^ Law Efficiency at MEPP
0.1	0.02	0.02
0.5	0.1	0.1
1	0.17	0.17
1.5	0.23	0.21
2	0.27	0.25
2.5	0.30	0.28
3	0.33	0.3
3.5	0.36	0.32
4	0.38	0.33
8	0.5	0.4
16	0.61	0.44
32	0.70	0.47
100	0.82	0.49

**Table 3:** Values of normalized electrical current $i_{\mathrm{MCEP},\mathrm{ep}}$, normalized thermal power $p_{\mathrm{th},\mathrm{MCEP}}$, and normalized electrical power $p_{\mathrm{el},\mathrm{MCEP}}$ at the MCEP in entropy pump mode for some hypothetical values of the figure-of-merit zT. Values of the second law power conversion efficiency can be read from Table 2

zT	$i_{\mathrm{MCEP},\mathrm{ep}}$	$p_{\mathrm{th},\mathrm{MCEP}}$	$p_{\mathrm{el},\mathrm{MCEP}}$
0.1	−20.49	41.95	1761.32
0.5	−4.45	9.80	97.01
1	−2.41	5.66	32.87
1.5	−1.72	4.22	19.67
2	−1.36	3.46	12.83
2.5	−1.48	2.99	10.77
3.0	−1	2.68	8.93
3.5	−0.89	2.42	7.56
4	−0.80	2.2	5.76
8	−0.50	1.5	3.00
16	−0.32	1.03	1.69
32	−0.21	0.71	1.02
100	−0.11	0.40	0.49

**Table 4:** Maximum figure-of-merit $zT_{\max}$ and corresponding power factor $\sigma \cdot \alpha^{2}$ of some state-of-the-art and emerging thermoelectric materials at temperature *T* with indication of conduction type.

Material	Type	${\mathrm{zT}}_{\max}$	$\sigma \cdot \alpha^{2}$	*T*	Ref.
		[μWcm${}^{-1}$K${}^{-2}$]	[K]		
(Bi${}_{0.25}$Sb${}_{0.75}$)${}_{2}$Te${}_{3}$	*p*	1.05	43	323	[70]
FeNb${}_{0.8}$Ti${}_{0.2}$Sb	*p*	1.10	53	973	[48,71]
Hf${}_{0.6}$Zr${}_{0.4}$Hf${}_{0.25}$NiSn${}_{0.995}$Sb${}_{0.005}$	*n*	1.20	47	900	[48,72]
Bi${}_{2}$(Te${}_{0.94}$Se${}_{0.06}$)${}_{3}$ (0.017 wt.% Te, 0.068 wt.% I)	*n*	1.25	57	298	[73]
(Bi${}_{0.25}$Sb${}_{0.75}$)${}_{2}$Te${}_{3}$ (8wt.% Te)	*p*	1.27	58	298	[73]
nano (Bi${}_{0.25}$Sb${}_{0.75}$)${}_{2}$Te${}_{3}$	*p*	1.4	38	373	[70]
ZrCoBi${}_{0.65}$Sb${}_{0.15}$Sn${}_{0.20}$	*p*	1.42	38	973	[48,74]
FeNb${}_{0.88}$Hf${}_{0.12}$Sb	*p*	1.45	51	1200	[48,75]
Bi${}_{0.88}$Ca${}_{0.06}$Pb${}_{0.06}$CuSeO	*p*	1.5	8	873	[48,76]
β-Cu${}_{2-x}$Se	*p*	1.5	12	1000	[77]
Ti${}_{0.5}$Zr${}_{0.25}$Hf${}_{0.25}$NiSn${}_{0.998}$Sb${}_{0.002}$Se	*n*	1.5	62	700	[48,78]
Mg${}_{3}$Sb${}_{1.48}$Bi${}_{0.4}$Te${}_{0.04}$	*n*	1.65	13	725	[79]
Ba${}_{0.08}$La${}_{0.05}$Yb${}_{0.04}$Co${}_{4}$Sb${}_{12}$	*n*	1.7	51	850	[80]
Mg${}_{3.175}$Mn${}_{0.025}$Sb${}_{1.5}$Bi${}_{0.49}$Te${}_{0.01}$	*n*	1.71	20	700	[48,81]
B-doped Si${}_{80}$Ge${}_{20}$ + YSi${}_{2}$	*p*	1.81	39	1073	[48,82]
Cu${}_{2-y}$S${}_{1/3}$Se${}_{1/3}$Te${}_{1/3}$	*p*	1.9	8	1000	[83]
AgPb${}_{m}$SbTe${}_{2+m}$	*n*	2.2	11	800	[84]
PbTe${}_{0.7}$S${}_{0.3}$-2.5%K	*p*	2.2	14	923	[68]
PbTe-4%SrTe-2%Na	*p*	2.2	24	915	[85]
Ge${}_{0.89}$Sb${}_{0.1}$In${}_{0.01}$Te	*p*	2.3	37	650	[86]
PbTe-8%SrTe	*p*	2.5	30	923	[87]
SnSe single crystal’s *b*-axis	*p*	2.6	10	923	[88]
β-Cu${}_{2}$Se/CuInSe${}_{2}$ (1% In)	*p*	2.6	12.5	850	[89]
SnSe${}_{0.97}$Br${}_{0.03}$ single crystal’s *a*-axis	*n*	2.8	9	773	[90]

**Figure A1:**
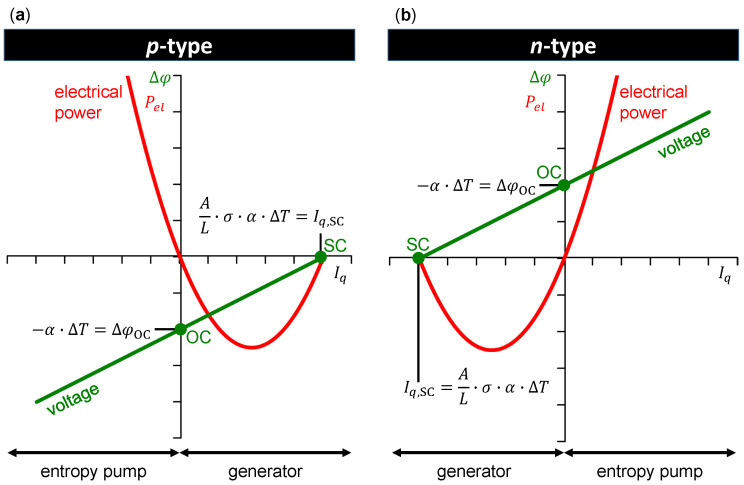
Voltage Δφ – electrical current $I_{q}$ characteristics (green curves) and electrical power $P_{\mathrm{el}}$ – electrical current characteristics $I_{q}$ (red curves) for materials with: (**a**) Seebeck coefficient α being positive, which refers to *p*-type conduction and (**b**) Seebeck coefficient α being negative, which refers to *n*-type conduction. Here, $\Delta T=\frac{T_{\mathrm{hot}}-T_{\mathrm{cold}}}{T_{\mathrm{hot}}}$ is the temperature difference along a thermoelectric material of length *L* and cross-sectional area *A*. These quantities, together with the (isothermal) electrical conductivity σ and the Seebeck coefficient, determine the electrical current $I_{\mathrm{SC}}$ under electrical short-circuited conditions. The voltage $\Delta \varphi_{\mathrm{OC}}$ under electrical short-circuited conditions is determined by the Seebeck coefficient and the temperature difference. When the electrical power $P_{\mathrm{el}}$ is negative (electrical power output), the material is in generator mode (thermal-to-electrical power conversion). When the electrical power $P_{\mathrm{el}}$ is positive (electrical power input), the material is in entropy pump mode (electrical-to-thermal power conversion).

**Figure A2:**
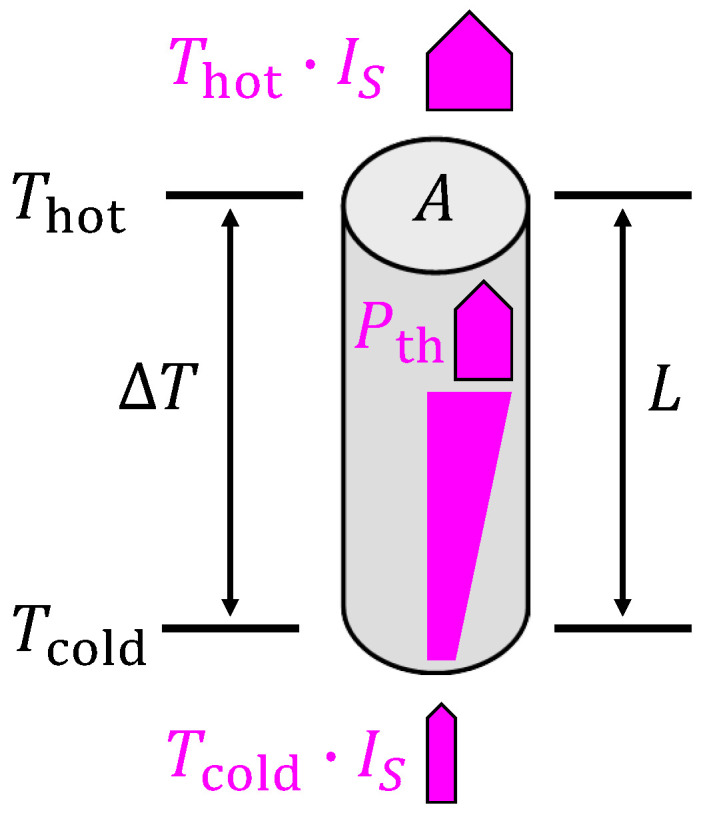
When the thermoelectric material is operated in entropy pump mode, electrical power $P_{\mathrm{el}}$, which is available by the fall of electric charge along Δφ, drives the pumping of entropy from the cold side to hot side. The thermal power $P_{\mathrm{th}}=\Delta T\cdot I_{S}=T_{\mathrm{hot}}\cdot I_{S}-T_{\mathrm{cold}}\cdot I_{S}$ for lifting entropy along the temperature difference ΔT adds to the thermal power removed from the cold side $T_{\mathrm{cold}}\cdot I_{S}$ to give the thermal power released to the hot side $T_{\mathrm{hot}}\cdot I_{S}$. Different width of arrows refers to different magnitudes of thermal power at the opposite sides of the material, which is due to thermoelectric power conversion.

## Abbreviations

The following abbreviations are used in this manuscript:

ECIP	Entropy Conductivity Inversion Point
MCEP	Maximum Conversion Efficiency Point (either in generator mode or entropy pump mode)
MEPP	Maximum Electrical Power Point (in generator mode)
OC	(Electrical) Open Circuit
SC	(Electrical) Short Circuit
**Symbols**	
The following symbols are used in this manuscript:	
*Geometry*	
*A*	cross-sectional area of thermoelectric material
*L*	length of thermoelectric material
*Material properties*	
α	Seebeck coefficient
*f*	figure-of-merit (as proposed by Zener [67])
λ	“heat” conductivity
$\lambda_{\mathrm{OC}}$	“heat” conductivity under electrically open-circuited (OC) conditions
Λ	entropy conductivity
$\Lambda_{\mathrm{OC}}$	entropy conductivity under electrically open-circuited (OC) conditions
$\Lambda_{\mathrm{SC}}$	entropy conductivity under electrically open-circuited (SC) conditions
$\tilde{\Lambda }$	normalized entropy conductivity
$M_{22}$	tensor element (of the thermoelectric material tensor)
*R*	electrical resistance (of thermoelectric material)
σ	isothermal electrical conductivity
*z*	thermoelectric factor (as introduced by Ioffe [56])
zT	figure-of-merit (as introduced by Ioffe [56])
$zT_{\max}$	maximum figure-of-merit
*Thermodynamic potentials*	
μ	chemical potential
$\tilde{\mu }$	electrochemical potential ($\tilde{\mu }=\mu +q\cdot \varphi$)
$\nabla \tilde{\mu }$	gradient of the electrochemical potential
$\nabla \tilde{\mu }/q$	gradient of the electrochemical potential per electric charge ($\nabla \tilde{\mu }/q=\nabla \mu /q+\nabla \varphi$)
φ	electrical potential
∇φ	gradient of the electrical potential
Δφ	difference of electrical potential (along the thermoelectric material)
$\Delta \varphi_{\mathrm{OC}}$	voltage under electrically open-circuited (OC) conditions
*T*	absolute temperature
$T_{\mathrm{cold}}$	temperature of the thermoelectric material at its cold side
$T_{\mathrm{hot}}$	temperature of the thermoelectric material at its hot side
∇T	gradient of the temperature
ΔT	difference of temperature (along the thermoelectric material)
*u*	normalized voltage
$u_{\mathrm{MEPP}}$	normalized voltage at the maximum electrical power point (MEPP)
*Fluxes*	
*A*	cross-sectional area of thermoelectric material
*L*	length of thermoelectric material
*i*	normalized electrical current
$i_{\mathrm{MCEP},\mathrm{ep}}$	normalized electrical current at the maximum conversion efficiency point (MCEP) in entropy pump mode
$i_{\mathrm{MCEP},\mathrm{gen}}$	normalized electrical current at the maximum conversion efficiency point (MCEP) in generator mode
$i_{\mathrm{MEPP}}$	normalized electrical current at the maximum electrical power point (MEPP)
$I_{q}$	electrical current
$I_{q,SC}$	electrical current at electrically short-circuited (SC) conditions
$I_{S}$	entropy current
$j_{q}$	electrical flux density
$j_{S}$	entropy flux density
*q*	electric charge
*S*	entropy
*Performance*	
${COP}_{\mathrm{cooler}}$	coefficient of performance of the thermoelectric material when used in a cooler
${COP}_{\mathrm{heater}}$	coefficient of performance of the thermoelectric material when used in a heater
$\eta_{I,\mathrm{gen}}$	first-law power conversion efficiency of the thermoelectric material in generator mode
$\eta_{\mathrm{II},\mathrm{gen}}$	second-law power conversion efficiency of the thermoelectric material in generator mode
$\eta_{\mathrm{II},\mathrm{gen},\max}$	maximum second-law power conversion efficiency of the thermoelectric material in generator mode
$\eta_{\mathrm{II},\mathrm{ep}}$	second-law power conversion efficiency of the thermoelectric material in entropy pump mode
$\eta_{\mathrm{II},\mathrm{ep},\max}$	maximum second-law power conversion efficiency of the thermoelectric material in entropy pump mode
$\eta_{C}$	Carnot’s efficiency
$p_{\mathrm{el}}$	normalized electrical power
$P_{\mathrm{el}}$	electrical power, needed for lifting electrical charge (generator mode)
	or made available by the fall of electric charge (entropy pump mode);
	simplified called output (generator mode) or input (entropy pump mode),
	when the electrical potential on one side of the thermoelectric material is set to zero
$P_{\mathrm{el},\;\max}$	maximum electrical power output of the thermoelectric material in generator mode (at the MEPP)
$P_{\mathrm{el},\mathrm{MCEP}}$	electrical power output, of the thermoelectric material in generator mode, at the MCEP
$P_{\mathrm{th}}$	thermal power, made available by the fall of entropy (generator mode)
	or needed for lifting entropy (entropy pump mode)

## Appendix A. Voltage–Electrical Current and Electrical Power–Electrical Current Characteristics: *p*- and *n*-Type Materials

The voltage–electrical current characteristics (green curve) and the electrical power–electrical current characteristics (red curve) of a thermoelectric material with either *p*-type or *n*-type conduction are given in Figure A1.

## Appendix B. Thermal-to-Electrical Power Conversion: Calculations and Established Models

### Appendix B.1. Maximum Electrical Power Point (MEPP): Material in Generator Mode

The MEPP is found by looking for the vanishing first derivative of the electrical power.

(A1) $$\begin{array}{ccc} 0 & =\frac{\partial P_{\mathrm{el}}}{\partial i} & =-\frac{A}{L}\cdot \sigma \cdot \alpha^{2}\cdot {\left(\Delta T\right)}^{2}\cdot \frac{\partial }{\partial i}\left(i-i^{2}\right)\\ \; & \; & =-\frac{A}{L}\cdot \sigma \cdot \alpha^{2}\cdot {\left(\Delta T\right)}^{2}\cdot \left(1-2\cdot i\right) \end{array}$$

The derivative vanishes if the term in the brackets vanishes, and the normalized current at the MEPP is as follows.

(A2) $$i_{\mathrm{MEPP}}=\frac{1}{2}$$

At the MEPP, the maximum electrical power is obtained as follows.

(A3) $$\begin{array}{ccc} P_{\mathrm{el},\max} & =P_{\mathrm{el}}\left(i_{\mathrm{MEPP}}\right) & =-\frac{A}{L}\cdot \sigma \cdot \alpha^{2}\cdot {\left(\Delta T\right)}^{2}\cdot \left(i_{\mathrm{MEPP}}-i_{\mathrm{MEPP}}^{2}\right)\\ \; & \; & =-\frac{A}{L}\cdot \sigma \cdot \alpha^{2}\cdot {\left(\Delta T\right)}^{2}\cdot \left(\frac{1}{2}-{\left(\frac{1}{2}\right)}^{2}\right)\\ \; & \; & =-\frac{A}{L}\cdot \sigma \cdot \alpha^{2}\cdot {\left(\Delta T\right)}^{2}\cdot \left(\frac{1}{2}-\frac{1}{4}\right)\\ \; & \; & =-\frac{1}{4}\cdot \frac{A}{L}\cdot \sigma \cdot \alpha^{2}\cdot {\left(\Delta T\right)}^{2} \end{array}$$

The 2^nd^-law power conversion efficiency at the MEPP is then obtained as follows.

(A4) $$\begin{array}{ccc} \eta_{\mathrm{II},\mathrm{gen},\mathrm{MEPP}} & =\eta_{\mathrm{II},\mathrm{gen}}\left(i_{\mathrm{MEPP}}\right) & =\frac{i_{\mathrm{MEPP}}-i_{\mathrm{MEPP}}^{2}}{i_{\mathrm{MEPP}}+\frac{1}{zT}}\\ \; & \; & =\frac{\frac{1}{2}-{\left(\frac{1}{2}\right)}^{2}}{\frac{1}{2}+\frac{1}{zT}}\\ \; & \; & =\frac{\frac{1}{2}-\frac{1}{4}}{\frac{1}{2}+\frac{1}{zT}}\\ \; & \; & =\frac{1}{4}\cdot \frac{1}{\frac{1}{2}+\frac{1}{zT}}\\ \; & \; & =\frac{zT}{4}\cdot \frac{1}{\frac{zT}{2}+1}\\ \; & \; & =\frac{1}{2}\cdot \frac{zT}{zT+2} \end{array}$$

### Appendix B.2. Maximum Conversion Efficiency Point (MCEP): Material in Generator Mode

The 2^nd^-law power conversion efficiency for a thermoelectric material operated in generator mode is obtained as follows.

(A5) $$\eta_{\mathrm{II},\mathrm{gen}}=|\frac{P_{\mathrm{el}}}{P_{\mathrm{th}}}|=\frac{\alpha \cdot I_{q}\cdot \Delta T-\frac{{I_{q}}^{2}}{\frac{A}{L}\cdot \sigma }}{\alpha \cdot I_{q}\cdot \Delta T+\frac{A}{L}\cdot \Lambda_{\mathrm{OC}}\cdot {\left(\Delta T\right)}^{2}}=\frac{\frac{I_{q}}{I_{q,\mathrm{SC}}}-{\left(\frac{I_{q}}{I_{q,\mathrm{SC}}}\right)}^{2}}{\frac{I_{q}}{I_{q,\mathrm{SC}}}+\frac{\Lambda_{\mathrm{OC}}}{\sigma \cdot \alpha^{2}}}$$

Substituting in Equation (A5) the dimensionless normalized electrical current $i=\frac{\left|I_{q}\right|}{\left|I_{q,\mathrm{SC}}\right|}$ and the figure-of-merit $zT=\frac{\sigma \cdot \alpha^{2}}{\Lambda_{\mathrm{OC}}}$, the 2^nd^ law power conversion efficiency can be written as follows.

(A6) $$\eta_{\mathrm{II},\mathrm{gen}}=\frac{i-i^{2}}{i+\frac{1}{zT}}$$

The maximum power conversion efficiency point (MCEP) can then be found by the first derivative to vanish.

(A7) $$\begin{array}{cc} 0 & =\frac{\partial \eta_{\mathrm{II},\mathrm{gen}}}{\partial i}\\ \; & =\frac{\partial }{\partial i}\left(\frac{i-i^{2}}{i+\frac{1}{zT}}\right)\\ \; & =\frac{\left(1-2\cdot i\right)\cdot \left(i+\frac{1}{zT}\right)-\left(i-i^{2}\right)\cdot 1}{{\left(i+\frac{1}{zT}\right)}^{2}}\\ \; & =\frac{i+\frac{1}{zT}-2\cdot i^{2}-2\cdot \frac{1}{zT}\cdot i-i+i^{2}}{{\left(i+\frac{1}{zT}\right)}^{2}}\\ \; & =\frac{-i^{2}-2\cdot \frac{1}{zT}\cdot i+\frac{1}{zT}}{{\left(i+\frac{1}{zT}\right)}^{2}} \end{array}$$

The derivative vanishes when the numerator vanishes.

(A8) $$i^{2}+2\cdot \frac{1}{zT}\cdot i-\frac{1}{zT}=0$$

This quadratic equation has two solutions, from which only one gives a positive-definite normalized current *i* at the maximum conversion efficiency point (MCEP).

(A9) $$\begin{array}{cc} i_{\mathrm{MCEP},\mathrm{gen}} & =\frac{\sqrt{1+zT}-1}{zT}\\ \; & =\frac{\left(\sqrt{1+zT}-1\right)\cdot \left(\sqrt{1+zT}+1\right)}{zT\cdot \left(\sqrt{1+zT}+1\right)}\\ \; & =\frac{1+zT+\sqrt{1+zT}-\sqrt{1+zT}-1}{zT\cdot \left(\sqrt{1+zT}+1\right)}\\ \; & =\frac{zT}{zT\cdot \left(\sqrt{1+zT}+1\right)}\\ \; & =\frac{1}{\sqrt{1+zT}+1} \end{array}$$

At the MCEP, the maximum power conversion efficiency of the thermoelectric material in generator mode is then obtained as follows.

(A10) $$\begin{array}{ccc} \eta_{\mathrm{II},\mathrm{gen},\max} & =\eta_{\mathrm{II},\mathrm{gen}}\left(i_{\mathrm{MCEP},\mathrm{gen}}\right) & =\frac{i_{\mathrm{MCEP},\mathrm{gen}}-i_{\mathrm{MCEP},\mathrm{gen}}^{2}}{i_{\mathrm{MCEP},\mathrm{gen}}+\frac{1}{zT}}\\ \; & \; & =i_{\mathrm{MCEP},\mathrm{gen}}\cdot \frac{1-i_{\mathrm{MCEP},\mathrm{gen}}}{i_{\mathrm{MCEP},\mathrm{gen}}+\frac{1}{zT}}\\ \; & \; & =i_{\mathrm{MCEP},\mathrm{gen}}\cdot \frac{\frac{1}{i_{\mathrm{MCEP},\mathrm{gen}}}-1}{1+\frac{1}{i_{\mathrm{MCEP},\mathrm{gen}}\cdot zT}}\\ \; & \; & =\frac{1}{\sqrt{1+zT}+1}\cdot \frac{\frac{1}{\frac{1}{\sqrt{1+zT}+1}}-1}{1+\frac{1}{\frac{1}{\sqrt{1+zT}+1}\cdot zT}}\\ \; & \; & =\frac{1}{\sqrt{1+zT}+1}\cdot \frac{\sqrt{1+zT}+1-1}{1+\frac{\sqrt{1+zT}+1}{zT}}\\ \; & \; & =\frac{1}{\sqrt{1+zT}+1}\cdot \frac{zT\cdot \sqrt{1+zT}}{zT+\sqrt{1+zT}+1}\\ \; & \; & =\frac{1}{\sqrt{1+zT}+1}\cdot \frac{zT\cdot \sqrt{1+zT}}{1+zT+\sqrt{1+zT}}\\ \; & \; & =\frac{1}{\sqrt{1+zT}+1}\cdot \frac{zT}{\sqrt{1+zT}+1}\\ \; & \; & =\frac{zT}{{\left(\sqrt{1+zT}+1\right)}^{2}}\\ \; & \; & =\frac{1+zT-1}{{\left(1+\sqrt{1+zT}\right)}^{2}}\\ \; & \; & =\frac{\left(\sqrt{1+zT}+1\right)\cdot \left(\sqrt{1+zT}-1\right)}{{\left(1+\sqrt{1+zT}\right)}^{2}}\\ \; & \; & =\frac{\sqrt{1+zT}-1}{\sqrt{1+zT}+1} \end{array}$$

By combining Equation (A9) and Equation (A10), the dependence of the maximum second-law efficiency on the electrical current can be shown to be linear.

(A11) $$\begin{array}{cc} \eta_{\mathrm{II},\mathrm{gen},\max}\left(i_{\mathrm{MCEP},\mathrm{gen}}\right) & =\frac{\sqrt{1+zT}-1}{\sqrt{1+zT}+1}\\ \; & =\frac{\sqrt{1+zT}+1-2}{\sqrt{1+zT}+1}\\ \; & =\frac{\frac{1}{i_{\mathrm{MCEP},\mathrm{gen}}}-2}{\frac{1}{i_{\mathrm{MCEP},\mathrm{gen}}}}\\ \; & =1-2\cdot i_{\mathrm{MCEP},\mathrm{gen}} \end{array}$$

The electrical power at the MCEP is as follows.

(A12) $$\begin{array}{ccc} P_{\mathrm{el},\mathrm{MCEP}} & =P_{\mathrm{el}}\left(i_{\mathrm{MCEP},\mathrm{gen}}\right) & =-\frac{A}{L}\cdot \sigma \cdot \alpha^{2}\cdot {\left(\Delta T\right)}^{2}\cdot \left(i_{\mathrm{MCEP},\mathrm{gen}}-i_{\mathrm{MCEP},\mathrm{gen}}^{2}\right)\\ \; & \; & =-\frac{A}{L}\cdot \sigma \cdot \alpha^{2}\cdot {\left(\Delta T\right)}^{2}\cdot \left(\frac{1}{\sqrt{1+zT}+1}-\frac{1}{{\left(\sqrt{1+zT}+1\right)}^{2}}\right)\\ \; & \; & =-\frac{A}{L}\cdot \sigma \cdot \alpha^{2}\cdot {\left(\Delta T\right)}^{2}\cdot \left(\frac{\sqrt{1+zT}+1}{{\left(\sqrt{1+zT}+1\right)}^{2}}-\frac{1}{{\left(\sqrt{1+zT}+1\right)}^{2}}\right)\\ \; & \; & =-\frac{A}{L}\cdot \sigma \cdot \alpha^{2}\cdot {\left(\Delta T\right)}^{2}\cdot \frac{\sqrt{1+zT}}{{\left(\sqrt{1+zT}+1\right)}^{2}}\\ \; & \; & =-\frac{1}{4}\cdot \frac{A}{L}\cdot \sigma \cdot \alpha^{2}\cdot {\left(\Delta T\right)}^{2}\cdot \frac{4\cdot \sqrt{1+zT}}{{\left(\sqrt{1+zT}+1\right)}^{2}}\\ \; & \; & =P_{\mathrm{el},\max}\cdot \frac{4\cdot \sqrt{1+zT}}{{\left(\sqrt{1+zT}+1\right)}^{2}} \end{array}$$

### Appendix B.3. Comparison to Power Conversion Efficiency after Fuchs: Thermogenerator Device

By accepting temperature and entropy as primitive quantities, Fuchs [33] has created aggregate dynamical models of a Peltier device. Suggesting the Peltier device to function analogously to a battery, he has derived linear voltage-electrical current characteristics and identified the only two dissipative processes, which are the diffusion of electric charge and the diffusion of entropy. For the case of the device being operated as a thermogenerator, Fuchs [33] has derived its 2^nd^-law efficiency by the ratio of useful to available power and expressed the efficiency with respect to the internal resistance of the device $R_{\mathrm{TEG}}$ and an external load resistance $R_{\mathrm{ext}}$.

(A13) $$\eta_{\mathrm{II},\mathrm{TEG}}=\frac{R_{\mathrm{ext}}}{R_{\mathrm{TEG}}+\left(R_{\mathrm{TEG}}+R_{\mathrm{ext}}\right)\cdot \frac{1}{zT}}\cdot \frac{R_{\mathrm{TEG}}}{R_{\mathrm{TEG}}+R_{\mathrm{ext}}}$$

For a given figure-of-merit zT, the 2^nd^-law efficiency of the device has its maximum at.

(A14) $$R_{\mathrm{ext}}=\sqrt{1+zT}\cdot R_{\mathrm{TEG}}$$

Thus, the maximum 2^nd^-law power conversion efficiency is as follows.

(A15) $$\begin{array}{cc} \eta_{\mathrm{II},\mathrm{TEG},\max} & =\frac{\sqrt{1+zT}\cdot R_{\mathrm{TEG}}}{R_{\mathrm{TEG}}+\left(R_{\mathrm{TEG}}+\sqrt{1+zT}\cdot R_{\mathrm{TEG}}\right)\cdot \frac{1}{zT}}\cdot \frac{R_{\mathrm{TEG}}}{R_{\mathrm{TEG}}+\sqrt{1+zT}\cdot R_{\mathrm{TEG}}}\\ \; & =\frac{\sqrt{1+zT}}{1+\left(1+\sqrt{1+zT}\right)\cdot \frac{1}{zT}}\cdot \frac{1}{1+\sqrt{1+zT}}\\ \; & =\frac{\sqrt{1+zT}}{zT+\left(1+\sqrt{1+zT}\right)}\cdot \frac{zT}{1+\sqrt{1+zT}}\\ \; & =\frac{\sqrt{1+zT}}{1+zT+\sqrt{1+zT}}\cdot \frac{zT}{1+\sqrt{1+zT}}\\ \; & =\frac{1}{\sqrt{1+zT}+1}\cdot \frac{zT}{1+\sqrt{1+zT}}\\ \; & =\frac{zT}{{\left(1+\sqrt{1+zT}\right)}^{2}}\\ \; & =\frac{1+zT-1}{{\left(1+\sqrt{1+zT}\right)}^{2}}\\ \; & =\frac{\left(\sqrt{1+zT}+1\right)\cdot \left(\sqrt{1+zT}-1\right)}{{\left(1+\sqrt{1+zT}\right)}^{2}}\\ \; & =\frac{\sqrt{1+zT}-1}{\sqrt{1+zT}+1} \end{array}$$

Of note, Fuchs has neglected the Joule “heat”, which would only have a small impact when the device is operated in generator mode. Note that Equation (A15) is equivalent to what has been obtained in this work for a thermoelectric material apart from a device (cf. Equation (A10)).

### Appendix B.4. Comparison to Power Conversion Efficiency after Altenkirch: Thermogenerator Device

Altenkirch [55] has estimated the power conversion efficiency for a thermogenerator (called thermopile at that time), which has been assumed to be made of two legs of dissimilar materials. For a small temperature difference along the device, which will cause only a small thermally-induced electrical current and allows neglect the Joule heating as well as the Thomson effect, he has derived his Equation (4) for the 1^st^-law power conversion efficiency. Altenkirch [55] has factorized the 1^st^ law power conversion efficiency into the Carnot efficiency and what we call here the 2^nd^-law power conversion efficiency $\eta_{\mathrm{II}}$. The latter has been of the following form.

(A16) $$\begin{array}{cc} \eta_{\mathrm{II},\mathrm{TEG}} & =\frac{zT\cdot \frac{R_{\mathrm{ext}}}{R_{\mathrm{TEG}}}}{zT\cdot \left(1+\frac{R_{\mathrm{ext}}}{R_{\mathrm{TEG}}}\right)+{\left(1+\frac{R_{\mathrm{ext}}}{R_{\mathrm{TEG}}}\right)}^{2}}\\ \; & =\frac{zT\cdot R_{\mathrm{ext}}\cdot R_{\mathrm{TEG}}}{zT\cdot \left(R_{\mathrm{TEG}}^{2}+R_{\mathrm{TEG}}\cdot R_{\mathrm{ext}}\right)+{\left(R_{\mathrm{TEG}}+R_{\mathrm{ext}}\right)}^{2}}\\ \; & =\frac{R_{\mathrm{ext}}\cdot R_{\mathrm{TEG}}}{R_{\mathrm{TEG}}\cdot \left(R_{\mathrm{TEG}}+R_{\mathrm{ext}}\right)+{\left(R_{\mathrm{TEG}}+R_{\mathrm{ext}}\right)}^{2}\cdot \frac{1}{zT}}\\ \; & =\frac{R_{\mathrm{ext}}}{R_{\mathrm{TEG}}+\left(R_{\mathrm{TEG}}+R_{\mathrm{ext}}\right)\cdot \frac{1}{zT}}\cdot \frac{R_{\mathrm{TEG}}}{R_{\mathrm{TEG}}+R_{\mathrm{ext}}} \end{array}$$

Here, Altenkirchs’s nomenclature has been substituted by $\frac{R_{\mathrm{ext}}}{R_{\mathrm{TEG}}}=x$ and $zT=10^{7}\cdot \eta^{\prime }$. In his treatment, the factor $10^{7}$ appeared due to the use of the calorie as the energy units, and “$\eta^{\prime }$” was called the effective thermopower of the device, which however contained the Seebeck coefficient multiplied with the square root of the ratio of specific thermal and specific electrical conductivities of the thermoelectric materials involved. Equation (A16) is equivalent to the result observed by Fuchs (cf. Equation (A13)).

Subsequently, Altenkirch derived the efficiency to be maximized for the following.

(A17) $$x=\frac{R_{\mathrm{ext}}}{R_{\mathrm{TEG}}}=\sqrt{1+zT}$$

Note that Equation (A17) is equivalent to the result obtained by Fuchs (cf. Equation (A14)).

For the thermoelectric generator (TEG), Altenkirch derived the maximum 2^nd^-law power conversion efficiency $\eta_{\mathrm{II},\mathrm{TEG},\max}$ to be (see Altenkirch [55], Equation (5)) as follows.

(A18) $$\begin{array}{cc} \eta_{\mathrm{II},\mathrm{TEG},\max} & =\frac{\sqrt{1+zT}-1}{\sqrt{1+zT}+1} \end{array}$$

Note that Equation (A18) is equivalent to the result obtained by Fuchs (cf. Equation (A15)).

Even though Altenkirch did not use the term figure-of-merit (compare Altenkirch [55], Figure 3), he plotted the maximum 2^nd^ law power conversion efficiency $\eta_{\mathrm{II},\mathrm{TEG},\max}$ as a function of $x=\frac{R_{\mathrm{ext}}}{R_{\mathrm{TEG}}}$ for different values of his “$\eta^{\prime }$”, which despite a dimensionless factor has been identified with zT. In the plot, he indicated the shift of the MCEP with varied figure-of-merit.

Altenkirch extended his approach by considering the impact of the Thomson effect on the power conversion efficiency. Moreover, he added remarks on the rate of thermal power exchange of the device with a hot reservoir and cold reservoir and its impact on the effective temperature difference along the device.

### Appendix B.5. Comparison to Power Conversion Efficiency after Ioffe: Thermogenerator Device

Ioffe [56] has considered a thermocouple in which legs of materials 1 and 2 of equal length are joined by a metallic bridge. The Seebeck coefficient of the device has been estimated from those of the two legs: $\alpha =|\alpha_{1}|+|\alpha_{2}|$. From equal length and the individual values of the electrical resistivities ($\rho_{1},\rho_{2}$), “heat” conductivities ($\lambda_{\mathrm{OC},1},\lambda_{\mathrm{OC},2}$) and cross-sectional area, he has calculated the total electrical resistance $R_{\mathrm{TEG}}$ and thermal conductance of the device $K_{\mathrm{TEG}}$ (see Ioffe [56], p. 36). To calculate the efficiency of thermal-to-electrical power conversion of the device, he has neglected the Thomson heat. Furthermore, he made an assumption regarding the Joule “heat” (see Ioffe [56], p. 38): “Of the total Joule ‘heat’ ${I_{q}}^{2}\cdot R_{\mathrm{TEG}}$ generated in the thermoelement, half passes to the hot junction, returning the power $\frac{1}{2}\cdot {I_{q}}^{2}\cdot R_{\mathrm{TEG}}$ and the rest is transferred to the cold junction.” As a result, the temperatures of the hot $T_{\mathrm{hot}}$ and cold junction $T_{\mathrm{cold}}$ appear in the maximum second-law efficiency.

(A19) $$\begin{array}{cc} \eta_{\mathrm{II},\mathrm{TEG},\max} & =\frac{\sqrt{1+z\bar{T}}-1}{\sqrt{1+z\bar{T}}+\frac{T_{\mathrm{hot}}}{T_{\mathrm{cold}}}} \end{array}$$

The aforementioned argument, which was probably inspired by Altenkirch’s [55] article, is based on misunderstanding the dissipation, which in the author’s opinion is thermal energy to leave the system together with produced entropy. The entropy, and thus the thermal energy, will not have driving force to flow to higher temperature. Anyway, following the argument, the thermal input power is diminished by half of the dissipated Joule “heat”. In this work, it has been outlined that the effect of Joule “heat” would be a diminished thermal power supply due to a changed temperature profile (cf. Section 4.2 in the the main text).

Neglecting the Joule “heat”, Ioffe has derived the following equation (see Ioffe [56], p. 40).

(A20) $$\begin{array}{cc} \eta_{\mathrm{II},\mathrm{TEG},\max} & =\frac{\sqrt{1+z\bar{T}}-1}{\sqrt{1+z\bar{T}}+1} \end{array}$$

Note that this is equivalent to what has been obtained in this work for a thermoelectric material apart from a device.

In the factor *z*, which Ioffe deduced (see Ioffe [56], p. 39), the cross-sectional areas $A_{1}$ and $A_{2}$ and length *L* cancel out, so it depends only on the thermoelectric properties of both materials but not their dimensions.

(A21) $$z=\frac{\alpha^{2}}{K_{\mathrm{TEG}}\cdot R_{\mathrm{TEG}}}=\frac{\alpha^{2}}{{\left(\sqrt{\lambda_{\mathrm{OC},1}\cdot \rho_{1}}+\sqrt{\lambda_{\mathrm{OC},2}\cdot \rho_{2}}\right)}^{2}}=\frac{\alpha^{2}}{{\left(\sqrt{\frac{\lambda_{\mathrm{OC},1}}{\sigma_{1}}}+\sqrt{\frac{\lambda_{\mathrm{OC},2}}{\sigma_{2}}}\right)}^{2}}$$

In the case that the electrical conductivities ($\sigma =\sigma_{n}=\sigma_{p}$) and “heat” conductivities ($\lambda_{\mathrm{OC}}=\lambda_{\mathrm{OC},1}=\lambda_{\mathrm{OC},2}$) are equal in both legs of the device, respectively, Equation (A21) becomes the following.

(A22) $$z=\frac{\sigma \cdot \alpha^{2}}{\lambda_{\mathrm{OC}}}$$

Ioffe used Equation (A22) when discussing a thermoelectric cooler (see Ioffe [56], p. 100) but derived an equivalent expression – using the thermal conductance instead of the thermal conductivity – when discussing the thermogenerator (see Ioffe [56], p. 38ff.). Anyway, in Equations (A19) and (A20) for the maximum power conversion efficiency, there appears not the factor *z* but this factor multiplied with the average temperature $\bar{T}$.

(A23) $$z\bar{T}=z\cdot \bar{T}=z\cdot \frac{T_{\mathrm{hot}}+T_{\mathrm{cold}}}{2}$$

Because of Ioffe’s Equations (A21)–(A23), the figure-of-merit of a thermoelectric material is currently termed $z\bar{T}$ or zT.

## Appendix C. Electrical-to-Thermal Power Conversion: Calculations and Established Models

### Appendix C.1. Power Conversion Efficiency

When the thermoelectric material is used in a cooler, the coefficient of performance COP is the ratio of the thermal power removed from the cold side $T_{\mathrm{cold}}\cdot I_{S}$ related to the electrical power $P_{\mathrm{el}}$.

(A24) $$\begin{array}{ccc} {COP}_{\mathrm{cooler}}=|\frac{T_{\mathrm{cold}}\cdot I_{S}}{P_{\mathrm{el}}}| & =|\frac{T_{\mathrm{cold}}\cdot I_{S}}{P_{\mathrm{th}}}|\cdot |\frac{P_{\mathrm{th}}}{P_{\mathrm{el}}}| & =\frac{T_{\mathrm{cold}}}{\Delta T}\cdot |\frac{P_{\mathrm{th}}}{P_{\mathrm{el}}}|\\ \; & \; & =\frac{T_{\mathrm{cold}}}{\Delta T}\cdot \frac{\frac{A}{L}\cdot \left(\Lambda_{\mathrm{OC}}+\sigma \alpha^{2}\cdot i\right)\cdot {\left(\Delta T\right)}^{2}}{\frac{A}{L}\cdot \sigma \cdot \alpha^{2}\cdot {\left(\Delta T\right)}^{2}\cdot \left(i-i^{2}\right)}\\ \; & \; & =\frac{T_{\mathrm{cold}}}{\Delta T}\cdot \frac{\frac{\Lambda_{\mathrm{OC}}}{\sigma \alpha^{2}}+i}{i-i^{2}}\\ \; & \; & =\frac{T_{\mathrm{cold}}}{\Delta T}\cdot \frac{i+\frac{1}{zT}}{-i^{2}+i}\\ \; & \; & =\frac{T_{\mathrm{cold}}}{\Delta T}\cdot \eta_{\mathrm{II},\mathrm{ep}} \end{array}$$

When the thermoelectric material is used in a heater, the coefficient of performance COP is the ratio of the thermal power released to the hot side $T_{\mathrm{hot}}\cdot I_{S}$ related to the electrical power $P_{\mathrm{el}}$.

(A25) $$\begin{array}{ccc} {COP}_{\mathrm{heater}}=|\frac{T_{\mathrm{hot}}\cdot I_{S}}{P_{\mathrm{el}}}| & =|\frac{T_{\mathrm{hot}}\cdot I_{S}}{P_{\mathrm{th}}}|\cdot |\frac{P_{\mathrm{th}}}{P_{\mathrm{el}}}| & =\frac{T_{\mathrm{hot}}}{\Delta T}\cdot |\frac{P_{\mathrm{th}}}{P_{\mathrm{el}}}|\\ \; & \; & =\frac{T_{\mathrm{hot}}}{\Delta T}\cdot \frac{i+\frac{1}{zT}}{-i^{2}+i}\\ \; & \; & =\frac{T_{\mathrm{hot}}}{\Delta T}\cdot \eta_{\mathrm{II},\mathrm{ep}}\\ \; & \; & =\frac{\eta_{\mathrm{II},\mathrm{ep}}}{\eta_{C}} \end{array}$$

The second-law efficiency for the thermoelectric material in entropy pump mode $\eta_{\mathrm{II},\mathrm{ep}}$ is as follows.

(A26) $$\begin{array}{ccc} \eta_{\mathrm{II},\mathrm{ep}} & =|\frac{P_{\mathrm{th}}}{P_{\mathrm{el}}}| & =\frac{\frac{A}{L}\cdot \left(\Lambda_{\mathrm{OC}}+\sigma \alpha^{2}\cdot i\right)\cdot {\left(\Delta T\right)}^{2}}{\frac{A}{L}\cdot \sigma \cdot \alpha^{2}\cdot {\left(\Delta T\right)}^{2}\cdot \left(i-i^{2}\right)}\\ \; & \; & =\frac{\frac{\Lambda_{\mathrm{OC}}}{\sigma \alpha^{2}}+i}{i-i^{2}}\\ \; & \; & =\frac{i+\frac{1}{zT}}{-i^{2}+i} \end{array}$$

The electrical power $P_{\mathrm{el}}$ used in Equations (A24)–(A26) is available by the fall of electric charge along the electrical potential difference Δφ. It drives the pumping of entropy from the material’s cold side to its hot side. The thermal power $P_{\mathrm{th}}=\Delta T\cdot I_{S}=T_{\mathrm{hot}}\cdot I_{S}-T_{\mathrm{cold}}\cdot I_{S}$ is needed for lifting entropy along the temperature difference ΔT. Some illustration is given in Figure A2.

### Appendix C.2. Maximum Conversion Efficiency Point (MCEP): Material in Entropy Pump Mode

The maximum power conversion efficiency point (MCEP) follows when the first derivative of the 2^nd^-law power conversion efficiency, as given by Equation (A26), vanishes.

(A27) $$\begin{array}{cc} 0 & =\frac{\partial \eta_{\mathrm{II},\mathrm{ep}}}{\partial i}\\ \; & =\frac{\partial }{\partial i}\left(\frac{i+\frac{1}{zT}}{-i^{2}+i}\right)\\ \; & =\frac{1\cdot \left(-i^{2}+i\right)-\left(i+\frac{1}{zT}\right)\cdot \left(-2\cdot i+1\right)}{{\left(-i^{2}+i\right)}^{2}}\\ \; & =\frac{-i^{2}+i+2\cdot i^{2}+\frac{2}{zT}\cdot i-i-\frac{1}{zT}}{{\left(-i^{2}+i\right)}^{2}}\\ \; & =\frac{i^{2}+\frac{2}{zT}\cdot i-\frac{1}{zT}}{{\left(-i^{2}+i\right)}^{2}} \end{array}$$

The derivative vanishes when the numerator vanishes.

(A28) $$\begin{array}{c} i^{2}+\frac{2}{zT}\cdot i-\frac{1}{zT}=0 \end{array}$$

The quadratic Equation (A28) has two solutions.

(A29) $$\begin{array}{cc} i_{1,2} & =-\frac{1}{zT}\pm \sqrt{{\left(\frac{1}{zT}\right)}^{2}+\frac{1}{zT}}\\ \; & =-\frac{1}{zT}\pm \frac{1}{zT}\cdot \sqrt{1+zT} \end{array}$$

From the two solutions shown in Equation (A29) only one fulfils the requirement $i\leq -\frac{1}{zT}$ for the material’s maximum conversion efficiency point (MCEP) in entropy pump mode. Thus, the normalized electrical current at the maximum conversion efficiency point (MCEP) is obtained as follows.

(A30) $$\begin{array}{cc} i_{\mathrm{MCEP},\mathrm{ep}} & =-\frac{1}{zT}-\frac{1}{zT}\cdot \sqrt{1+zT}\\ \; & =-\frac{1+\sqrt{1+zT}}{zT}\\ \; & =-\frac{\sqrt{1+zT}+1}{zT}\;!\\ \; & =-\frac{\sqrt{1+zT}+1}{1+zT-1}\\ \; & =-\frac{\sqrt{1+zT}+1}{\left(\sqrt{1+zT}+1\right)\cdot \left(\sqrt{1+zT}-1\right)}\\ \; & =-\frac{1}{\sqrt{1+zT}-1} \end{array}$$

The maximum 2^nd^-law power conversion efficiency for a thermoelectric material operated in entropy pump mode is then as follows.

(A31) $$\begin{array}{ccc} \eta_{\mathrm{II},\mathrm{ep},\max} & =\eta_{\mathrm{II},\mathrm{ep},\max}\left(i_{\mathrm{MCEP},\mathrm{ep}}\right) & =\frac{i_{\mathrm{MCEP},\mathrm{ep}}+\frac{1}{zT}}{-i_{\mathrm{MCEP},\mathrm{ep}}^{2}+i_{\mathrm{MCEP},\mathrm{ep}}}\\ \; & \; & =\frac{-\frac{\sqrt{1+zT}+1}{zT}+\frac{1}{zT}}{-{\left(\frac{\sqrt{1+zT}+1}{zT}\right)}^{2}-\frac{\sqrt{1+zT}+1}{zT}}\\ \; & \; & =\frac{zT}{zT}\cdot \frac{-\sqrt{1+zT}-1+1}{-\frac{1}{zT}\cdot {\left(\sqrt{1+zT}+1\right)}^{2}-\left(\sqrt{1+zT}+1\right)}\\ \; & \; & =\frac{1}{-\left(\sqrt{1+zT}+1\right)}\cdot \frac{-\sqrt{1+zT}}{\frac{1}{zT}\cdot \left(\sqrt{1+zT}+1\right)+1}\\ \; & \; & =\frac{zT}{\sqrt{1+zT}+1}\cdot \frac{\sqrt{1+zT}}{\left(\sqrt{1+zT}+1\right)+zT}\\ \; & \; & =\frac{zT}{\sqrt{1+zT}+1}\cdot \frac{\sqrt{1+zT}}{1+zT+\sqrt{1+zT}}\\ \; & \; & =\frac{zT}{\sqrt{1+zT}+1}\cdot \frac{1}{\sqrt{1+zT}+1}\\ \; & \; & =\frac{1+zT-1}{\sqrt{1+zT}+1}\cdot \frac{1}{\sqrt{1+zT}+1}\\ \; & \; & =\frac{\left(\sqrt{1+zT}+1\right)\cdot \left(\sqrt{1+zT}-1\right)}{\sqrt{1+zT}+1}\cdot \frac{1}{\sqrt{1+zT}+1}\\ \; & \; & =\frac{\sqrt{1+zT}-1}{\sqrt{1+zT}+1} \end{array}$$

By combining Equations (A30) and (A31), the dependence of the maximum second-law efficiency on the electrical current can be shown to be hyperbolic.

(A32) $$\begin{array}{cc} \eta_{\mathrm{II},\mathrm{ep},\max}\left(i_{\mathrm{MCEP},\mathrm{ep}}\right) & =\frac{\sqrt{1+zT}-1}{\sqrt{1+zT}+1}\\ \; & =\frac{\sqrt{1+zT}-1}{\sqrt{1+zT}-1+2}\\ \; & =\frac{\frac{-1}{i_{\mathrm{MCEP},\mathrm{ep}}}}{\frac{-1}{i_{\mathrm{MCEP},\mathrm{ep}}}+2}\\ \; & =\frac{1}{1-2\cdot i_{\mathrm{MCEP},\mathrm{ep}}} \end{array}$$

The normalized thermal power (cf. Appendix C.3) at the MCEP is obtained by combining Equations (A35) and (A30).

(A33) $$\begin{array}{ccc} p_{\mathrm{th},\mathrm{MCEP}} & =p_{\mathrm{th}}\left(i_{\mathrm{MCEP},\mathrm{ep}}\right) & =4\cdot |\frac{1}{zT}-\frac{1}{\sqrt{1+zT}-1}|\\ \; & \; & =\frac{4}{zT}\cdot |1-\frac{zT}{\sqrt{1+zT}-1}|\\ \; & \; & =\frac{4}{zT}\cdot |1-\frac{1+zT-1}{\sqrt{1+zT}-1}|\\ \; & \; & =\frac{4}{zT}\cdot |1-\frac{\left(\sqrt{1+zT}+1\right)\cdot \left(\sqrt{1+zT}-1\right)}{\sqrt{1+zT}-1}|\\ \; & \; & =\frac{4}{zT}\cdot |1-\left(\sqrt{1+zT}+1\right)|\\ \; & \; & =\frac{4}{zT}\cdot |1-\sqrt{1+zT}-1|\\ \; & \; & =\frac{4}{zT}\cdot |-\sqrt{1+zT}|\\ \; & \; & =4\cdot |-\frac{\sqrt{1+zT}}{zT}|\\ \; & \; & =4\cdot \frac{\sqrt{1+zT}}{zT} \end{array}$$

The absolute thermal power at the MCEP in entropy pump mode, which is related to the MEPP in generator mode, is thus the following:

(A34) $$\begin{array}{cc} |P_{\mathrm{th},\mathrm{MCEP}}| & =p_{\mathrm{th},\mathrm{MCEP}}\cdot P_{\mathrm{el},\max}\\ \; & =4\cdot \frac{\sqrt{1+zT}}{zT}\cdot P_{\mathrm{el},\max} \end{array}$$

### Appendix C.3. Normalized Thermal Power

The normalized thermal power $p_{\mathrm{th}}$ is obtained as follows.

(A35) $$\begin{array}{cc} p_{\mathrm{th}} & =\frac{|P_{\mathrm{th}}|}{P_{\mathrm{el},\max}}\\ \; & =\frac{|\frac{A}{L}\cdot \left(\Lambda_{\mathrm{OC}}+\sigma \alpha^{2}\cdot i\right)\cdot {\left(\Delta T\right)}^{2}|}{\frac{1}{4}\cdot \frac{A}{L}\cdot \sigma \cdot \alpha^{2}\cdot {\left(\Delta T\right)}^{2}}\\ \; & =4\cdot |\frac{\Lambda_{\mathrm{OC}}}{\sigma \alpha^{2}}+i|\\ \; & =4\cdot |\frac{1}{zT}+i| \end{array}$$

### Appendix C.4. Comparison to Power Conversion Efficiency after Altenkirch: Thermoelectric Cooler Device

For a thermoelectric cooler made of two legs of dissimilar thermoelectric materials (called a thermopile) in steady-state condition, Altenkirch [57] has derived an expression for the minimum electrical power input related to a given cooling power (see Altenkirch [57], Equation (12)), which factorizes into a Carnot-type factor $\frac{T_{\mathrm{hot}}-T_{\mathrm{cold}}}{T_{\mathrm{cold}}}$ and the reciprocal of what he called the dissipation factor for the electro-thermal device. It must be emphasized that the Carnot-type factor introduced by Altenkirch is different from Carnot’s efficiency because it relates the temperature difference $T_{\mathrm{hot}}-T_{\mathrm{cold}}$ to the temperature of the cold side $T_{\mathrm{cold}}$ instead of the hot side $T_{\mathrm{hot}}$. This is due to the thermal energy current removed from the cold side being related to the electrical power input.

When Altenkirch’s nomenclature is substituted by $zT=10^{7}\cdot \eta^{\prime }$, his dissipation factor (see Altenkirch [57], Equation (13)) for the thermoelectric cooler (TEC), which is the device-related analogue of what we here call the maximum 2^nd^-law power conversion efficiency for a thermoelectric material operated in entropy pump mode $\eta_{\mathrm{II},\mathrm{ep},\max}$, becomes as follows.

(A36) $$\begin{array}{cc} \eta_{\mathrm{II},\mathrm{TEC},\max} & =\frac{\sqrt{1+zT}-\frac{T_{\mathrm{hot}}}{T_{\mathrm{cold}}}}{\sqrt{1+zT}+1} \end{array}$$

Altenkirch [57] states that, for small temperature differences (i.e., $\frac{T_{\mathrm{hot}}}{T_{\mathrm{cold}}}\approx 1$), the maximum 2^nd^-law power conversion efficiency for thermoelectric cooler $\eta_{\mathrm{II},\mathrm{TEC},\max}$ becomes the following.

(A37) $$\begin{array}{cc} \eta_{\mathrm{II},\mathrm{TEC},\max} & =\frac{\sqrt{1+zT}-1}{\sqrt{1+zT}+1} \end{array}$$

Altenkirch’s result of Equation (A37) for a device is identical to the maximum 2^nd^-law power conversion efficiency for a thermoelectric material operated in entropy pump mode $\eta_{\mathrm{II},\mathrm{ep},\max}$ as obtained in this work (see Equation (A31)).

### Appendix C.5. Comparison to Power Conversion Efficiency after Ioffe: Thermoelectric Cooler Device

For a thermoelectric cooler made of two legs of dissimilar thermoelectric materials, Ioffe [56] (see Ioffe [56], p. 99) has derived a maximum coefficient of performance COP, which he factorized into the inverse of a Carnot-type factor $\frac{T_{\mathrm{cold}}}{T_{\mathrm{hot}}-T_{\mathrm{cold}}}$ and what we here call the maximum 2^nd^-law efficiency $\eta_{\mathrm{II},\mathrm{ep},\max}$. After Ioffe [56], the device-related analogue of the latter has been as follows.

(A38) $$\begin{array}{cc} \eta_{\mathrm{II},\mathrm{TEC},\max} & =\frac{\sqrt{1+\frac{1}{2}\cdot z\cdot \left(T_{\mathrm{hot}}+T_{\mathrm{cold}}\right)}-\frac{T_{\mathrm{hot}}}{T_{\mathrm{cold}}}}{\sqrt{1+\frac{1}{2}\cdot z\cdot \left(T_{\mathrm{hot}}+T_{\mathrm{cold}}\right)}+1} \end{array}$$

In the case of small temperature difference (i.e., $\frac{T_{\mathrm{cold}}}{T_{\mathrm{hot}}}\approx 1$) and when identifying the average temperature $T=\frac{1}{2}\cdot \left(T_{\mathrm{hot}}+T_{\mathrm{cold}}\right)$, it becomes identical to the result of this work for a thermoelectric material (see Equation (A31)).
